# ADAR1-mediated RNA editing promotes B cell lymphomagenesis

**DOI:** 10.1016/j.isci.2023.106864

**Published:** 2023-05-12

**Authors:** Riccardo Pecori, Weicheng Ren, Mohammad Pirmoradian, Xianhuo Wang, Dongbing Liu, Mattias Berglund, Wei Li, Rafail Nikolaos Tasakis, Salvatore Di Giorgio, Xiaofei Ye, Xiaobo Li, Annette Arnold, Sandra Wüst, Martin Schneider, Karthika-Devi Selvasaravanan, Yvonne Fuell, Thorsten Stafforst, Rose-Marie Amini, Kristina Sonnevi, Gunilla Enblad, Birgitta Sander, Björn Engelbrekt Wahlin, Kui Wu, Huilai Zhang, Dominic Helm, Marco Binder, F. Nina Papavasiliou, Qiang Pan-Hammarström

**Affiliations:** 1 Division of Immune Diversity (D150), German Cancer Research Center (DKFZ), Heidelberg, Germany; 2 Helmholtz Institute for Translational Oncology (HI-TRON), Mainz, Germany; 3 Department of Biosciences and Nutrition, Karolinska Institutet, Stockholm, Sweden; 4 Department of Lymphoma, Tianjin Medical University Cancer Institute and Hospital, National Clinical Research Center of Cancer, Key Laboratory of Cancer Prevention and Therapy, Tianjin, China; 5 BGI-Shenzhen, Shenzhen, China; 6 Guangdong Provincial Key Laboratory of Human Disease Genomics, Shenzhen Key Laboratory of Genomics, Shenzhen, China; 7 Department of Immunology, Genetics and Pathology, Uppsala University, Uppsala, Sweden; 8 Graduate Program in Biosciences, University of Heidelberg, Heidelberg, Germany; 9 Research Group "Dynamics of Early Viral Infection and the Innate Antiviral Response", Division Virus-Associated Carcinogenesis (F170), German Cancer Research Center (DKFZ), Heidelberg, Germany; 10 Proteomics Core Facility (W120), German Cancer Research Center (DKFZ), Heidelberg, Germany; 11 Interfaculty Institute of Biochemistry, University of Tübingen, Tübingen, Germany; 12 Hematology Unit, Department of Medicine, Huddinge, Karolinska Institutet and Medical Unit Hematology, Karolinska University Hospital, Solna, StockholmSweden; 13 Department of Laboratory Medicine, Karolinska University Hospital, Huddinge, Stockholm, Sweden

**Keywords:** Genetics, Molecular biology, Epigenetics, Omics, Transcriptomics

## Abstract

Diffuse large B cell lymphoma (DLBCL) is one of the most common types of aggressive lymphoid malignancies. Here, we explore the contribution of RNA editing to DLBCL pathogenesis. We observed that DNA mutations and RNA editing events are often mutually exclusive, suggesting that tumors can modulate pathway outcomes by altering sequences at either the genomic or the transcriptomic level. RNA editing targets transcripts within known disease-driving pathways such as apoptosis, p53 and NF-κB signaling, as well as the RIG-I-like pathway. In this context, we show that ADAR1-mediated editing within *MAVS* transcript positively correlates with MAVS protein expression levels, associating with increased interferon/NF-κB signaling and T cell exhaustion. Finally, using targeted RNA base editing tools to restore editing within *MAVS* 3′UTR in ADAR1-deficient cells, we demonstrate that editing is likely to be causal to an increase in downstream signaling in the absence of activation by canonical nucleic acid receptor sensing.

## Highlights

• RNA editing targets transcripts within DLBCL disease-driving pathways
• RNA editing may represent a mechanism complementary to that of DNA mutation
• *MAVS* editing directly correlates with its protein levels and downstream signaling
• Targeted restoration of editing within *MAVS* restores downstream pathway signaling

## Graphical abstract

**Figure:**
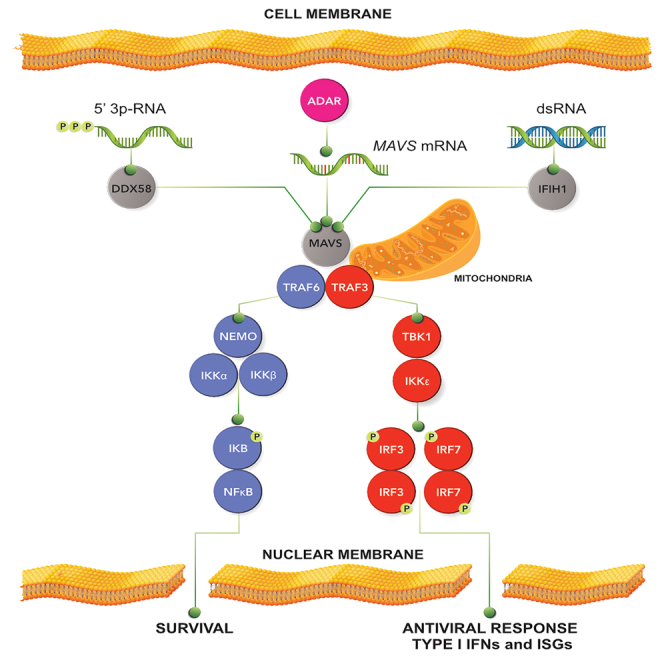

## Introduction

Diffuse large B cell lymphoma (DLBCL) is one of the most common and aggressive types of lymphoma. Based on gene expression profiling, two major “cell-of-origin” subtypes of DLBCL have been identified, referred to as germinal center B cell-like (GCB) and activated B cell-like (ABC), respectively.^1^ In recent years, high-throughput genomic studies further identified recurrent genetic alterations within the key disease-driving genes/pathways in DLBCL*,* such as *BCL6* and *MYC*, epigenetic modifiers, p53/DNA damage response pathway, B cell receptor (BCR)/NF-κB-, NOTCH- and JAK-STAT-signaling pathways as well as genes related to antigen presentation and immune evasion.^3^^,^^4^^,^^5^^,^^6^^,^^7^^,^^8^ Based on these genetic alterations, several molecular subtypes have been suggested.^4^^,^^6^^,^^8^^,^^9^ Nevertheless, the disease heterogeneity of DLBCL cannot be fully explained by the genetic changes, both regarding the biological nature of the cancers and their response to treatment.^10^

Although DNA alterations (mutations or more extensive structural changes such as large deletions, duplications and translocations) or gene expression changes can be critical to tumor initiation, maintenance and progression, post-transcriptional RNA modifications together with altered RNA binding protein activity (the ‘epitranscriptome’) have recently been characterized as contributors to clonal evolution in cancer, through dynamic determination of RNA function and gene expression in response to environmental stimuli.^12^^,^^13^^,^^14^^,^^15^^,^^16^ Of all epitranscriptomic modifications recently described, RNA editing is the most common and straightforward to catalog and validate. RNA editing occurs by hydrolytic deamination of cytosine (C) to uracil (U) (mediated by the activation-induced cytidine deaminase (AID)/APOBEC family of proteins) or of adenosine (A) to inosine (I) (decoded as guanosine, mediated by the ADAR family of proteins), which cause respectively thymine (T) or guanine (G) base changes in cDNA, as compared to the genomic reference^16^ (hence the term “editing”). Next-generation sequencing approaches have been particularly adept at identifying inconsistencies in base-pair composition between the genomic sequence (DNA) and its corresponding transcribed RNA (cDNA), which are then referred to as putative RNA editing events. Based on such approaches, it has become clear that RNA editing is rampant and mediates specific functional outcomes depending on where it occurs on the transcript: coding region editing, although rare, can alter decoding and thus protein sequence^17^; whereas the far more common editing identified within non-coding regions can affect splicing, RNA stability, transcript localization and translational efficiency (and thus the general composition of protein levels within cells).^18^^,^^19^

In contrast to genomic mutations, which represent the historical archive of insults to the DNA, RNA editing is an active mark: If the editing enzyme is inhibited or lost, editing disappears, and the transcriptome reverts to faithfully mirroring the expressed genome of the cell. However, an unedited transcriptome is also a rich source of dsRNAs, and acute loss of ADAR1 leads to inflammation and cell death in healthy tissue through the accumulation of unedited endogenous dsRNA and consequent activation of corresponding sensing pathways.^21^^,^^22^^,^^23^^,^^24^ Hypomorphic mutations in *ADAR1* were observed in patients with Aicardi-Goutières syndrome, which is characterized by uncontrolled interferon (IFN) expression and secretion^24^ and belongs to a group of autoinflammatory disorders referred to as type I interferonopathies.^25^ In the context of tumor tissues, it has recently been shown that general loss of ADAR1 (and of editing) causes cell-intrinsic lethality through endogenous dsRNA mediated induction of IFN, a key anti-tumor cytokine, and subsequently to robust inflammation of the tumor microenvironment (with substantial tumor infiltration by CD8^+^T cells) and increased response to immune checkpoint inhibitors.^27^^,^^28^^,^^29^

Here, we profile the RNA editing events and assess the role of ADAR1 in immune evasion in DLBCL. We used matched DNA and RNA sequencing datasets derived from DLBCL tumor biopsies to precisely determine the transcripts that are edited in DLBCL and the signaling pathways they represent. We observed that specific pathways associated with disease progression can be altered either by mutation and/or by editing – an unexplored concept in lymphomagenesis, which we then validated using targeted base editing to perturb the specific transcripts (*MAVS*, encoding mitochondrial antiviral signaling protein), on an editing-null background. We also assess the role of ADAR1-mediated *MAVS* editing in mediating inflammation through the induction of IFN/NF-κΒ, and find that a lower level of ADAR1 or MAVS in B cell lymphoma does not correlate with an increase of IFN response as observed for solid tumors (such as lung adenocarcinoma or pancreatic cancer^29^). Increased level of ADAR1 or MAVS is, however, associated with increased T cell exhaustion. Overall, ADAR1-mediated RNA editing represents an additional mechanism underlying B cell lymphomagenesis, and our findings have implications for ADAR1-centered immunotherapies currently under development.

## Results

### Robust ADAR1 mediated RNA editing in DLBCL

To explore the role of ADARs RNA editing in DLBCL, we first profiled 106 DLBCL samples, using matched genomic (in the form of whole genome or whole exome sequencing) and transcriptomic sequencing data (Table S1). Then, we applied a recently developed machine learning-based method to identify specific RNA editing sites^30^ to our dataset (Figure S1). This classifier, named RED-ML, was built on carefully constructed positive and negative training sets, and can efficiently remove possible sequencing artifacts, misalignments during analysis, as well as genomic mutations^30^ (for details also see the material availability and STAR Methods section). In addition, paired DNA sequencing was used to further filter out any DNA mutation present in the dataset, substantially reducing the possibility that RNA editing events called through this pipeline would inadvertently include true DNA mutations. Using these pipelines, we identified 57610 RNA editing sites across all DLBCL samples. The vast majority of the editing sites (97%) identified in our dataset have already been reported to the REDIPORTAL database^31^ (Figure S1C), a rigorously annotated database of RNA editing, supporting the reliability of our RNA editing detection pipeline. Furthermore, we have validated the identified RNA editing sites using matched RNA and genome DNA samples derived from the same tumor biopsies by Sanger sequencing and cloning in selected genes (*CARD11* and *TP73*) (Figure S2).

**Figure 1:**
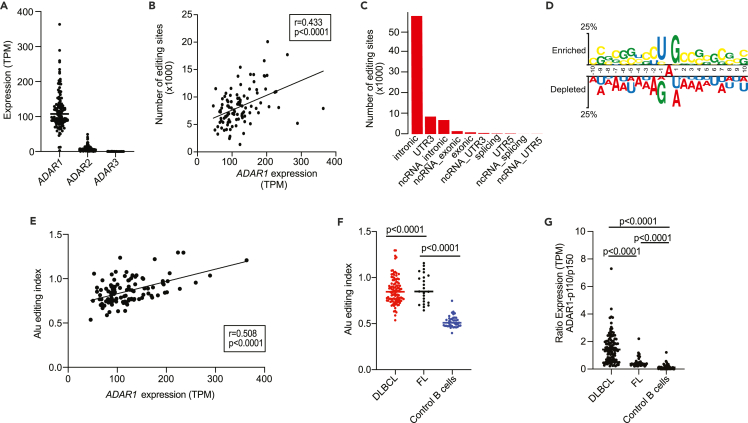
Robust ADAR1 catalyzed RNA editing in DLBCL (A) *ADAR1* expression levels in a cohort of 106 DLBCL patients suggests that ADAR1 is the main adenosine deaminase active in these tumors. (B) Within tumors, increased *ADAR1* expression is correlated with the number of editing sites identified from RED-ML (p<0.0001). (C and D) RNA editing in DLBCL shows features consistent with ADAR1 activity: most A-to-I editing involves Alu repeats within non-coding regions (intronic or 3′UTR) within specific sequence features. (E) Within tumors, increased *ADAR1* expression is correlated with increased AEI (p<0.0001). (F) The comparison of AEI in DLBCL, FL and control B cells. (G) An increase in p110 in DLBCL (and concomitant decrease of p150) versus FL or control B cells, leads to an increased ADAR1 p110/p150 ratio that is characteristic of DLBCL. For panels B and E, Pearson correlation coefficient was used for r and p values. For panels F and G, p values were calculated using Mann-Whitney U test.

Consistent with previous reports, the most abundant editing type was A-to-I (A-to-G) (reviewed in Eisenberg and Levanon 2018^16^) (Figure S1D), and we further focused on these dominant editing events. The human genome encodes three ADARs: ADAR1 is ubiquitously expressed and, together with ADAR2, are catalytically active, whereas ADAR3 is catalytically inactive and acts as an inhibitor of editing.^32^^,^^33^ Initial comparison of the expression level of each of the three ADARs encoded in the human genome suggested ADAR1 as the main adenosine editor in DLBCL (Figure 1A). Furthermore, *ADAR1* expression positively correlated with the total number of editing sites per patient (r = 0.43, p < 0.0001; Figure 1B) and 90% A-to-I editing involved Alu repeats within non-coding regions (i.e., introns and 3′UTRs) (Figures 1C, and S1D), consistent with previous reports on ADAR1 activity.^35^^,^^36^^,^^37^^,^^38^ Moreover, motif analysis of these sites showed preferential usage of U and G, as 5′- and -3′ neighbor bases of the edited A, as known flanking sequence preference of ADAR1 (Figure 1D).^39^^,^^40^^,^^41^ In addition, the edited transcripts were not biased for transcript length, GC content or expression level (Figure S3), in line with previous reports.^35^^,^^37^^,^^42^^,^^43^^,^^44^

As a control, we performed the same analysis on a previously published dataset from EBV immortalized peripheral blood B cells from 41 healthy individuals (referred to as control B cells),^45^^,^^46^^,^^47^ where only 17,858 RNA editing sites were identified. To allow unbiased comparison between samples/cohorts, we next broadly quantified ADAR1-mediated RNA editing activity by calculating the Alu editing index (AEI).^47^ *ADAR1* expression showed an even stronger positive correlation with the AEI per DLBCL patient (r = 0.508, p < 0.0001; Figure 1E) as compared to that with the total number of editing sites (Figure 1B). Using this approach, we further confirmed that DLBCL samples have significantly increased editing compared to control B cells (p < 0.0001, Figure 1F). Similarly, increased level of editing was also observed in another type of B cell lymphoma, follicular lymphoma (FL; n = 20; Figure 1F), suggesting the increase of editing as a more general feature for germinal center related B-cell malignancies (see also *ref.*^48^). Altogether these data demonstrate the presence of robust A-to-I ADAR1 mediated RNA editing in DLBCL with sequence features similar to those identified for other types of cancers.^11^^,^^49^

Increased expression of ADAR1 in cancer is often assumed to refer to overexpression of ADAR1 p150, the interferon-driven ADAR1 isoform.^50^^,^^51^ Unexpectedly, we observed that in DLBCL, increased ADAR1 expression is instead closely correlated with increased expression of the ubiquitous isoform of ADAR1, p110. Since p110 is also the nuclear form responsible for the majority of editing, this explains the high level of editing in these tumors (Figure 1F). Indeed, the higher ratio of ADAR1 p110/p150 is not only significant in DLBCL versus FL or control B cells (Figure 1G) but is also associated with a late disease stage (Table S2).

### A landscape for RNA editing in DLBCL

**Figure 2:**
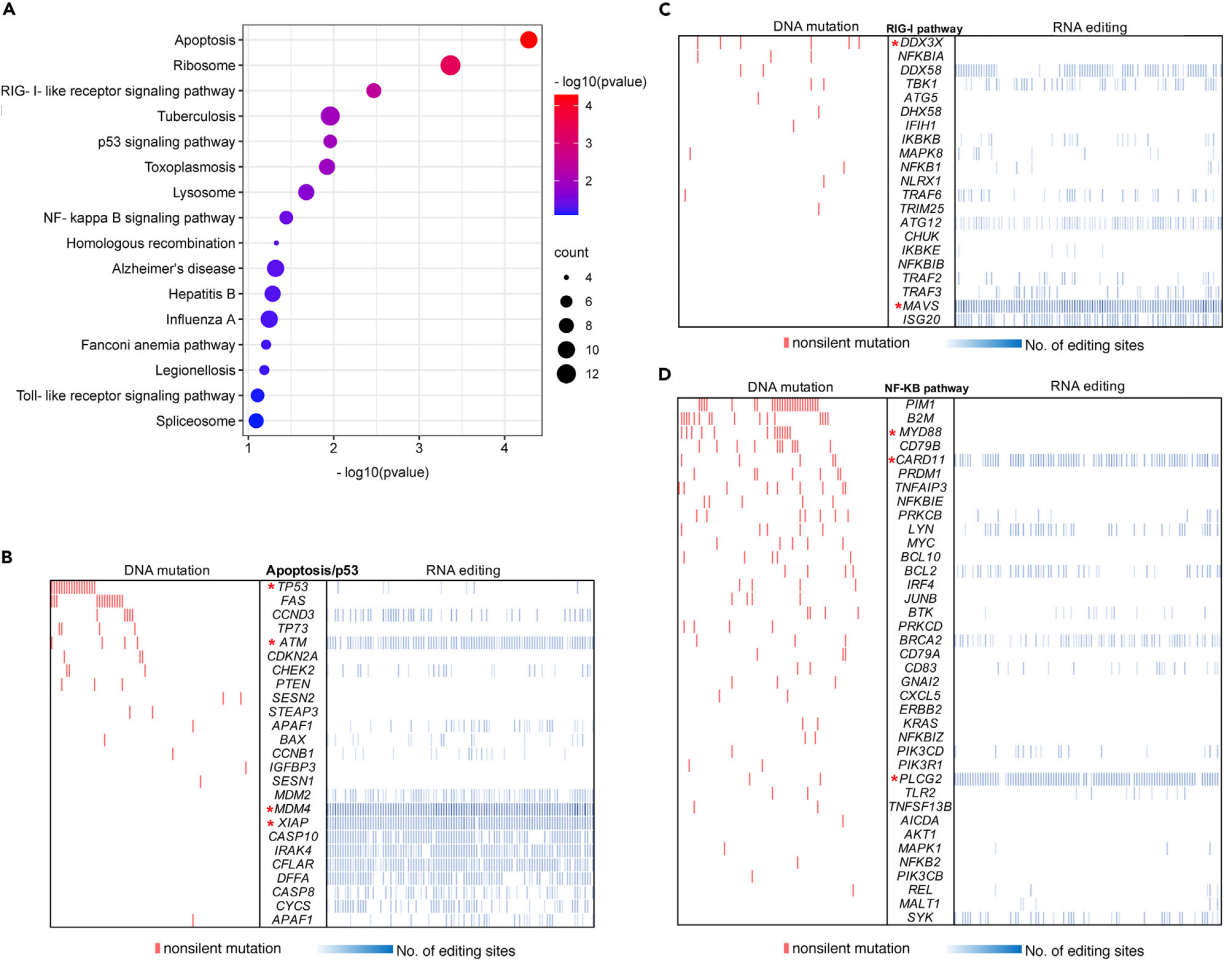
The landscape of RNA editing versus DNA mutation in DLBCL (A) KEGG pathway enrichment of genes affected by unique RNA editing sites identified in DLBCL but absent in control B cells. Only the editing sites identified in functional regions were considered in the analysis, and the editing sites identified in more than 10% of DLBCL but absent in control B cells. (B–D) RNA editing versus DNA mutation. Genes that belong to the Apoptosis/p53 pathway (B), the RIG-I like Receptor (RLR) pathway (which usually culminates in interferon signaling in response to viral infection) (C) and the NF-κB signaling pathway (of key relevance to lymphoma progression) (D) are shown in the figure. These pathways can be perturbed either by editing (right) or by mutation (left). For example, *TP53* is mutated in ∼30% of the patients (red bars), but *ATM* is edited in almost all patients, as is *MDM4* (a regulator of p53 activity). Each column represents a patient, and each row a gene. The p value in panel A was calculated using Fisher’s exact test. Genes mentioned in the results session are highlighted by a red star (∗).

To begin to characterize the role of ADAR1-mediated editing in DLBCL, we concentrated on A-to-I RNA editing sites that can be assigned to a gene locus and that were enriched in DLBCL patient samples. For this, we first excluded all editing occurring in non-genic regions and then cataloged editing sites occurring in more than 10% of DLBCL patient samples (but which were absent from the transcriptomes of control B cell samples). This analysis yielded 4605 unique editing sites present in 834 genes (Tables S3A and S3B). Furthermore, pathway analysis using the Kyoto Encyclopedia of Genes and Genomes (KEGG) database suggested that these edited genes were for pathways of relevance to DLBCL (like the apoptosis/p53 signaling pathway or the NF-κB signaling pathway - Figure 2A) but also for pathways that have not been associated with DLBCL (like the RIG-I-like Receptor (RLR) signaling pathway; Figure 2A).

Delving deeper into the genes that define these pathways, we assessed how many of these were mutated at the DNA level or were found edited at the RNA level. We observed that genes that belong to the apoptosis/p53 pathway (like *ATM, XIAP* and *MDM4*), or genes which belong to the NF-κB pathway (such as *PLCG2* and *CARD11)* or finally genes within the RLR pathway (which culminates in IFN signaling in response to viral infection) were often found either edited or mutated (Figures 2B–2D). For example, *TP53* was mutated in ∼30% of the patients (red bars) but only occasionally edited (blue bars), whereas *ATM* was heavily edited but infrequently mutated (Figure 2B). Another example in the apoptosis/p53 signaling pathway is *MDM4* (encoding a regulator of p53 activity – Figure 2B), which was edited in almost all samples, but not mutated. In the RLR pathway, *DDX3X* was only mutated but not edited in DLBCL, whereas *MAVS* was never mutated but heavily edited (Figure 2C). Similarly, in the NF-κB pathway, *MYD88* was only mutated, whereas other genes such as *CARD11* and *PLCG2* were more often edited (Figure 2D). Finally, altered editing frequencies did not arise from differences in mRNA expression levels for the genes of interest, between DLBCL samples and control (non-malignant) B cells (Figure S3). These results highlight that RNA editing occurs within transcripts belonging to pathways necessary for DLBCL survival and maintenance, and within those pathways and with few exceptions, editing and mutation appear to be mutually exclusive (Table S4, Figure S4). This aspect suggests that functionally, RNA editing may represent a mechanism complementary to that of DNA mutation.

### ADAR1 editing of *MAVS* is correlated with increased MAVS protein levels and increased downstream signaling

Narrowing down from transcriptome-level editing (specified by the AEI) to gene-level editing in the context of pathways affected (Figures 2B–2D), the RLR pathway was identified as a potentially unrecognized candidate disease-driving pathway for DLBCL. In the current view of RLR pathway signaling, the signal consists of the deposition of viral RNA within the cytoplasm, which is sensed by the cytoplasmic RNA sensors RIG-I (*DDX58*) or MDA5 (*IFIH1*). Once these sensors bind the viral dsRNA, they interact with the MAVS protein on the mitochondrial membrane and nucleate the formation of MAVS polymers. The switch from soluble MAVS to self-propagating helical MAVS fibers (reminiscent of amyloid and prions) is thought to activate and amplify downstream signaling^52^ through cytosolic protein kinases of the IKK family, which in turn activate the transcription factors NF-κB and IRF3. Activated NF-κB and IRF3 subsequently translocate to the nucleus, where they induce the expression of type I IFN and other inflammatory molecules (Figure S5A and *ref.* ^21^). Because sensing through the RLR pathway culminates in NF-κB and type I IFN signalings (important for tumor cell survival or inflammation in the tumor microenviroment), we further investigated a possible role of ADAR1-mediated RNA editing in the activation of this pathway.

**Figure 3:**
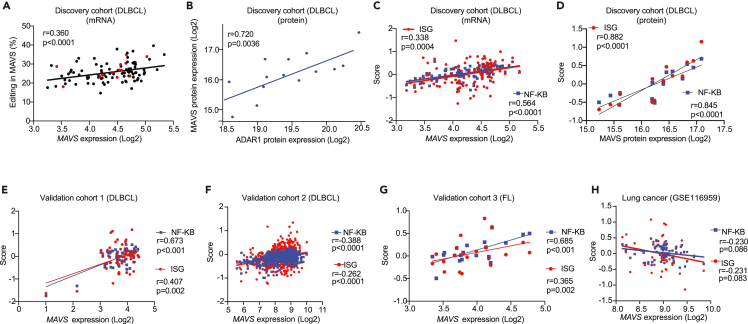
ADAR1 editing of *MAVS* is correlated with increased MAVS protein levels and increased downstream signaling (A) Correlation between *MAVS* editing and *MAVS* expression (p<0.0001, Pearson correlation coefficients). (B) Proteomic data for n = 14 samples (corresponding tumors are marked in red in 3A) show a correlation between MAVS and ADAR1 protein levels (p = 0.0036, Pearson correlation coefficients). (C) *MAVS* expression and correlation with ISG (in red)/NF-κB (in blue) scores (p values are p = 0.0004 for ISG and p<0.0001 for NF-κB respectively). (D) MAVS protein expression and correlation with ISG (in red)/NF-κB (in blue) scores in the protein level (p values are p<0.0001 for both ISG and NF-κB). (E and F) *MAVS* expression and correlation with ISG (in red)/NF-κB (in blue) scores in two different and independent DLBCL validation cohorts. validation cohort 1 (n = 54), a Swedish DLBCL cohort. validation cohort 2 (n = 420), GSE10846. (G) *MAVS* expression and correlation with ISG (in red)/NF-κB (in blue) scores in a third independent FL validation cohort (n = 20, p values are p = 0.002 for ISG and p<0.001 for NF-κB respectively). (H) *MAVS* expression and correlation with ISG (in red)/NF-κB (in blue) scores in lung adenocarcinoma (p values are p = 0.083 for ISG and p = 0.086 for NF-κB respectively). Pearson correlation coefficient was used for r and p values.

In DLBCL samples and within the RLR pathway, *MAVS* is not mutated, but it is highly edited (Figure 2C). Of interest, *MAVS* editing is positively correlated with increased transcript levels (Figure 3A). To assess whether MAVS protein levels were affected by editing, we performed mass spectrometry-based proteomic analyses on a small subset of DLBCL samples (n = 14, noted as red dots in expression data in Figure 3A). Within these randomly selected samples, we could identify a positive correlation of ADAR1 and MAVS protein levels (Figure 3B). These data together demonstrate that ADAR1-mediated RNA editing is positively correlated with an increase in *MAVS* mRNA and also in MAVS protein levels in the absence of additional, exogenous signaling (e.g., viral infections associated with DLBCL, including HBV and EBV –Figure S6). We further showed that MAVS expression, both at the transcript and the protein level, is positively correlated with the ISG/NF-κB gene expression scores (Figures 3C and 3D). We validated these findings in two independent DLBCL cohorts (validation cohorts 1 and 2, Figures 3E and 3F), as well as the FL samples (validation cohort 3; Figure 3G); in contrast, a negative correlation was observed in a lung adenocarcinoma cohort (Figure 3H). Notably, unlike *DDX58* (*RIG-I*) or *IFIH1* (*MDA5*), *MAVS* itself is not an ISG (*ref.*^53^ and Figure S7), and overexpression of MAVS is sufficient to trigger pathway activation.^53^^,^^54^ We, therefore, hypothesize that robust ADAR1-mediated editing of the *MAVS* transcript observed in DLBCL patients (Figure 2B) would provide an alternative, and RLR-independent way of signaling toward ISG/NF-κB pathway activation (Figure S5B).

### Targeted restoration of editing on *MAVS* on a background of zero editing directly recapitulates increased downstream signaling

To test the hypothesis that *MAVS* editing might lead to increased downstream signaling, we first analyzed the location of A-to-I RNA editing within the *MAVS* transcript from DLBCL versus control B cells. All RNA editing sites reported are within the 3′UTR of the transcript and can be grouped in six clusters of short “hyper-edited” regions surrounded by relatively unedited stretches (Figure S8). Although many editing sites are present in both DLBCL samples and control B cells, most editing events within clusters 3 and 4 are specific to DLBCL (Figure S8B and Table S3A) and are also present within a DLBCL-derived cell line RC-K8 (Figures S8B–S8D). We thus proceeded to knockout ADAR1 from RC-K8 cells using the CRISPR/Cas9 technology (Figure S9). This substantially reduced editing (Figure S9E) but did not abolish it: all three targeted clones we obtained show deletions in the proximity of the sgRNA sites resulting in loss of exons 3 and 4, at the cDNA and protein level (Figures S9B–S9F); this deleted version of ADAR1 retains residual RNA editing activity. Despite this, total RNA editing, quantified as AEI, is substantially reduced (especially in clone 18, which we then used in restoration experiments) (Figure S9E). Comparing RNA editing sites detected from RC-K8 wildtype versus knockout RNA-seq data, we could confirm the absence or reduction of all editing sites within *MAVS* – demonstrating that they are ADAR1-dependent (Figure S8C). Finally, we also validated a few sites using RT-PCR and Sanger sequencing (Figure S8D).

**Figure 4:**
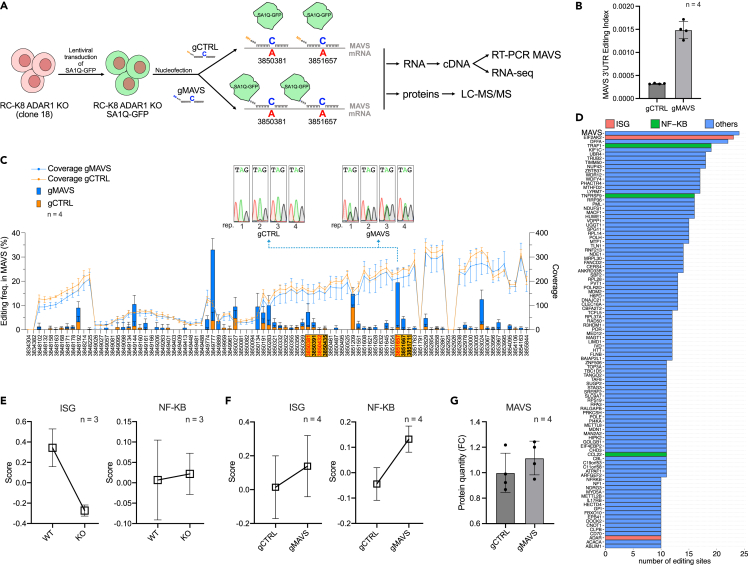
Base editor dependent re-targeting of the *MAVS* 3′UTR modulates the inflammatory output of the NF-κB and type-I IFN signaling cascades (A) Cartoon of the SNAP-ADAR1 targeted editing system used to induce RNA editing within *MAVS* 3′UTR. RC-K8 KO (clone 18) was transduced to express SA1Q-GFP stably. This cell line was then nucleofected with two chemically stabilized bisbenzylguanine(BB)-modified guide RNAs (BB-gRNAs or gMAVSs) or without BB group (NH-gRNAs or gCTRLs). 24h later, RNA and protein were extracted, and RT-PCR on MAVS, RNA-seq, and mass spectrometry analysis were performed. (B) The bar plot represents the editing index calculated for the 3′UTR of MAVS in the presence of gMAVSs or gCTRLs. (C) Editing frequency per position along the 3′UTR of MAVS. The bars indicate the frequency of editing in each position in the presence of gMAVSs or gCTRLs. The coverage per position is shown by overlapping dot plots. (D) Barplot representing the 100 genes with the highest number of significantly edited editing sites within their transcripts in gMAVS samples in comparison to gCTRL samples. ISGs and NF-κB genes are labeled in red and green, respectively. (E and F) A comparison of ISG and NF-κB scores between ADAR1 WT and KO RC-K8 cells (F) A comparison of ISG and NF-κB scores between ADAR1 KO RC-K8 cells in which MAVS editing is restored (gMAVS) and the relevant controls (gCTRL), indicates increased signaling on MAVS editing restoration. (G) MAVS protein quantification via mass spectrometry in the same samples. n = number of independent biological replicates. For panels B, C and G, data are represented as mean ± SD.

To test whether ADAR1-mediated editing of *MAVS* 3′UTR is a direct and RLR-independent mechanism of activating canonical MAVS signaling, we used a recently described targeted base editing system^55^ to re-introduce editing specifically onto the *MAVS* 3′UTR in ADAR1-“deficient” RC-K8 cells. This system uses an engineered mutant of the DNA repair protein O^6^-alkylguanine-DNA alkyltransferase, called SNAP-tag, which is able to form a covalent bond with O^6^-benzylguanine (BG),^56^ conjugated to the deaminase domain of ADAR1 carrying the E406Q mutation typical of the hyperactive variants^57^ and GFP (SA1Q-GFP). First, the RC-K8 ADAR1 KO cells with the lowest level of ADAR1 expression and AEI (clone 18 – Figures S9B–S9D) were transduced for stable expression of SA1Q-GFP (Figure 4A). Second, we designed two chemically stabilized bisbenzylguanine(BB)-modified guide RNAs (BB-gRNAs or gMAVSs) to target simultaneously clusters 3 and 4 within *MAVS* at multiple sites and restore ADAR1-mediated RNA editing in these DLBCL-specific regions (Figure S10). As a control, we used the same gRNAs before the addition of BB group (NH-gRNAs or gCTRLs), leading to a gRNA able to create a dsRNA but not to recruit SA1Q-GFP allowing us to discriminate between SA1Q-GFP and unspecific editing (Figures 4A, and S10). Using these strategies, we observed specific gMAVS-dependent editing at the targeted location via RT-PCR (Figure S11A). However, within the targeted transcript, editing was not necessarily site-specific. Indeed, RNA editing analysis from RNA-seq showed restoration of editing in several sites along the 3′UTR of *MAVS*, altogether leading to a higher editing index for this region in the presence of gMAVSs compared to gCTRLs (Figures 4B and 4C). Thus, selective loading of the enzymes onto a transcript can broadly affect editing within a region flanking the targeted sites. Unexpectedly, we also observed a considerable difference in the editing measured from RNA-seq data or RT-PCR and Sanger sequencing (Figures 4C, S11A), suggesting that the guide-dependent editing on the 3′UTR of *MAVS* is possibly higher than what measured from RNA-seq (resulting in reads that are unmappable, as previously suggested^58^). Finally, we analyzed the ability of gMAVSs to generate off-target editing events. Despite the underestimation of editing in *MAVS* by RNA-seq, we found the highest number of statistically significant editing sites within its 3′UTR (Figure 4D, Supplementary File 1). In addition, this editing leads to a small but measurable increase in MAVS transcript levels (Figure S11B), recapitulating the correlation seen in Figure 3A. Altogether, the restoration of editing with gMAVSs was reasonably specific, on par with the best previously published data using this system.^55^

To then assess our hypothesis that restoration of *MAVS* editing could lead to increased downstream signaling (as predicted by correlative data in Figure 3), we calculated the ISG and NF-κB scores based on the transcriptomic data from the RC-K8 ADAR KO cells transfected with SA1Q-GFP and gMAVSs or gCTRLs. This analysis demonstrated that targeted RNA base editing in *MAVS* could restore (or increase) levels of ISGs and of NF-κB responsive genes, which were reduced (or unaffected) in the ADAR1 knockout cells (Figures 4E and 4F). In line with our hypothesis, expression of MAVS protein was reduced in RC-K8 ADAR1 KO cells (Figure S11D), whereas MAVS protein levels increased on targeted editing, leading to the increase in ISG and NF-κB scores (Figures 4F and 4G). However, we also detected many off-target editing events in the presence of gMAVS (Figure S11C). To evaluate if off-target editing could affect ISG and NF-κB signaling, we identified the top 100 transcripts containing the highest number of differentially edited editing sites (Figure 4D) and only a minority of those (5%) are ISGs or NF-κB genes (Figure 4D), strengthening the notion that the increased ISG/NF-κB scores most likely resulted specifically from the restoration of editing on MAVS.

Overall, these data demonstrate that targeted editing of the untranslated region (the 3′UTR) of a key gene within a pathway may be sufficient to modulate the output of that pathway. More specifically, these results support the hypothesis that ADAR1-mediated *MAVS* editing can lead to increased MAVS protein levels and to increased, non-canonical downstream signaling, especially through NF-κB, a key oncogenic signaling pathway in DLBCL pathogenesis. These findings are in line with a recent publication showing that MAVS protein level is affected by ADAR3 through inhibition of ADAR1 editing on *MAVS* 3′UTR.^59^ Furthermore, these data demonstrate that ADAR1-mediated *MAVS* editing also leads to increased ISGs expression in B cell malignancies, which is an unusual observation, given that reduction or loss of ADAR1 in most other studies on solid tumors is associated with activation of inflammatory cascades, as indicated by the ISG signatures, rather than their silencing^21^^,^^22^^,^^26^^,^^27^^,^^29^^,^^60^ and Figure S9G-I, S12A-B). Altogether, these data reveal an alternative, and RLR-independent way of signaling toward ISG/NF-κB via RNA editing.

### In DLBCL, MAVS expression is associated with T cell exhaustion within a relatively uninflamed tumor microenvironment

**Figure 5:**
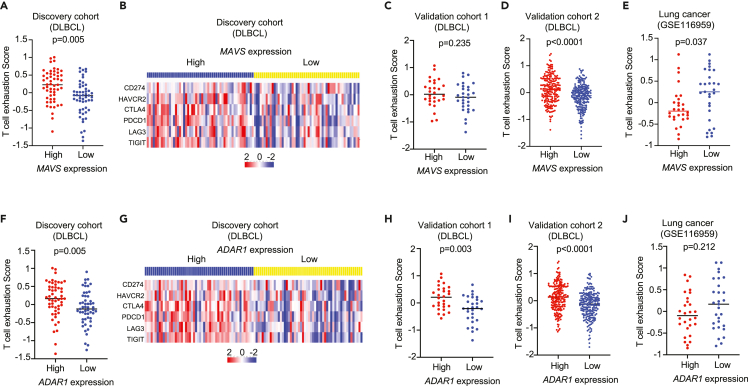
*MAVS*/*ADAR1* expression within DLBCL tumor cells is a biomarker for T cell exhaustion within a relatively uninflamed TME The expression of 6 genes was used to measure the T cell exhaustion in tumors by using RNA-seq data. (A–E) The analysis of T cell exhaustion in patients with *MAVS* high (top 50%) and low (bottom 50%) expression in different cohorts, including DLBCL discovery cohort (n = 106, A-B), DLBCL validation cohort 1 (n = 54, Swedish DLBCL cohort, C), validation cohort 2 (n = 420, GSE10846, D), as well as lung cancer cohort (n = 57, GSE116959, E). (F–J) The analysis of T cell exhaustion in patients with *ADAR1* high (top 50%) and low (bottom 50%) expression in different cohorts, including DLBCL discovery cohort (F-G), DLBCL validation cohort 1 (n = 54, Swedish DLBCL cohort, H), validation cohort 2 (n = 420, GSE10846, I), as well as lung cancer cohort (n = 57, GSE116959, J). B and G is a heatmap showing the expression of 6 genes used to calculate T cell exhaustion scores in the respective groups. For panels A, C-F, and H-J, bars indicated the mean of each group and p values were calculated by the Mann-Whitney U test.

Recent work has shown that loss of ADAR1 from tumors inflames the tumor microenvironment (TME) through ISG upregulation, consequently leading to substantial infiltration by CD8^+^T cells.^27^ Here, we have shown that *MAVS* expression (modulated at least in part through ADAR1-mediated *MAVS* editing) is positively correlated with the ISG/NF-κB scores in DLBCL (Figures 3C and 3D). Therefore, a change in *MAVS* expression (because of ADAR1 editing) within the tumor seems to be a strong corollary of ISG/NF-κB signaling and presumably of downstream interferon production and tumor survival signaling, respectively. We then asked how *MAVS* expression would correlate with an influx of cells within the TME consistent with an increase in inflammation. We used the TIMER tool^61^ to predict the composition of the tumor-infiltrating immune cells between tumors that expressed high levels of *MAVS* (top 50%) versus those that showed low *MAVS* expression (bottom 50%). Within this analysis, we did not find substantial changes, especially T cells in TME composition relating to the expression of *MAVS* (Figure S13), in contrast to what has been reported for solid tumors^27^ and consistent with our observation here, i.e., in B cell lymphomas, expression of *MAVS* positively correlated with ISG/NF-κB scores (Figures 3C–3F). However, when T cell functional features from the DLBCL tumors were further evaluated, we found that high expression of *MAVS* is significantly correlated with increased T cell exhaustion signature in DLBCLs (Figure 5A and 5B). Similar trends were observed in the two independent DLBCL cohorts (Figures 5C and 5D), but a negative correlation was noted in lung cancers (Figure 5E). Similar results were obtained when comparing the *ADAR1* high- and low-expressing tumors (Figures 5F–5J). Taken together, our data suggest that *MAVS* expression (or ADAR1-mediated *MAVS* editing) in DLBCL (as opposed to other solid tumors such as lung cancer) is positively correlated with inflammation cascade and exhaustion status of the tumor-infiltrating T cells. In addition, our data imply that DLBCL tumors with high *MAVS* expression (through increased editing) might be associated with a worse prognosis, and this is certainly a trend in our data with a significantly higher score of the international prognostic index (IPI) in MAVS high versus low group (Table S5). Beyond this, our data suggest that different types of tumors can benefit from general ADAR1 inhibition (or perhaps specific inhibition of *MAVS* editing) via different mechanisms, i.e., either through an increase in inflammation within the TME (for solid tumors) or through a decrease in T cell exhaustion (for B cell lymphoma). Thus, *MAVS* editing can potentially be a better biomarker than ISG scores,^26^ in the context of selecting tumors for therapy with ADAR1 inhibitors.

## Discussion

Aberrant inflammation is an emerging hallmark of tumorigenesis. In that regard, pattern recognition receptors (PRRs) which are essential mediators of the host immune response, are also critical elements affecting tumor pathobiology, with the Toll-like receptor (TLR) or Nod-like receptor (NLR) pathways critically involved in cancer.^62^ A third, the RLR pathway, has yet to be evaluated in the context of cancer.

Signaling through the RLR pathway is tightly regulated, with activation usually through the sensing of viral nucleic acids (through sensors such as RIG-I, MDA5 or even TLR3) and suppression through a set of negative regulators, one of which is the RNA editing enzyme ADAR1. Under homeostatic conditions, aberrant activation of the RLR pathway is prevented by editing endogenous dsRNA by ADAR1. Consequently, loss of ADAR1 results in the induction of IFN, a key anti-tumour cytokine, and subsequently to robust inflammation of the tumor microenvironment (substantial tumor infiltration by CD8^+^T cells) and increased response to tumor restriction by checkpoint inhibitors.^27^ Although this is the case for three types of solid tumors studied (melanoma, lung adenocarcinoma and acinar pancreatic carcinoma,^26^^,^^27^^,^^29^^,^^60^ we discovered that in DLBCL, one of the most common and aggressive types of B cell lymphomas, reduction or loss of ADAR1 was irrelevant to key proximal measures of inflammation within the tumor microenvironment (Figure 5). Thus, the regulation of the RLR pathway in B cells, which are part of the adaptive immune system, and their malignant counterparts, is most likely different from other types of non-immune cells. This statement is further supported by our analyses in DLBCL derived cell lines (like RC-K8) which are capable of responding to stimulation through the RLR pathway (Figure S12C) and also by recent reports of an alternative pathway to MDA5/MAVS that can rescue the inflammatory phenotype mediated by loss of ADAR1 in mature immune cells^63^ (and which could be preferentially expressed in B cells or other cells of the immune system). It is also worth noting that while loss of MAVS is well tolerated in solid tumor derived cell lines (e.g. HCC366^26^), it has a moderate anti-proliferation effect on B cells (Figure S14, and *ref.*^64^), again favoring the notion that the regulation of the RLR pathway in B cell derived tumors, is distinct.

Focusing on the RLR pathway, a potential pathway in the context of cancer in general and DLBCL in particular, we find that *MAVS*, the central RLR adaptor molecule, while not mutated, is robustly edited (Figure 2C), with editing correlated with increased gene and protein expression levels and increased downstream signaling (Figure 3). But why would MAVS translation be promoted when the transcript is better edited? The MAVS 3′UTR contains six Alu repeats, 4 of which are direct repeats. Direct Alu repeats have been correlated with transcript localization to the cytoplasm followed by increased translation,^65^ whereas inverted repeats are correlated with increased nuclear retention. It is possible that the balance toward direct Alu repeats encoded in the MAVS 3′UTR it the molecular cause for the observed increase in translation.

MAVS is known to aggregate in response to ligand (viral RNA) bound RIG-I (*DDX58*) or MDA5 (*IFIH1*); however, viral infection did not correlate with MAVS (or ADAR1) levels in DLBCL: even though some of the samples showed evidence of viral infection (illustrated by HBV^+^ or EBV^+^ status), the editing rate (as defined by the AEI index) was not altered (Figure S6). We hypothesized that MAVS aggregation on protein overexpression subsequent to RNA-editing of the *MAVS* 3′UTR (but in the absence of a canonical upstream ligand) would be sufficient to increase downstream signaling. To directly assess this we used specific RNA base-editing technologies to target RNA editing to the *MAVS* 3′UTR on an ADAR1 deficient background, revealing an alternative, RLR-independent way of signaling toward ISG/NF-κB via RNA editing. This is, also beyond the context of DLBCL, a demonstration that RNA editing of single or several sites within a 3′UTR can activate a downstream signaling pathway.

We hypothesize that the outcome of the RLR signaling pathway is modulated not only by *MAVS* editing but also by editing of additional components, including additional RLR components. Overall, we propose that specific patterns of editing in DLBCL (represented by the DLBCL cell line RC-K8) lead to activation to the NF-κB branch of the RLR pathway supporting tumor survival signaling. Finally, MAVS editing/signaling is not associated with the number of T cells infiltrated in the TME but is associated with the T cell exhaustion status, suggesting a possible specific immune evasion mechanism for DLBCL.

Overall, the experiments reported here demonstrate specific functions for ADAR1 in DLBCL that are distinct from those described for solid tumors and have implications for ADAR-centered immunotherapies currently under development.

### Limitations of the study

The main limitation of this study concerns the technical characteristics of targeted RNA-base editing approaches. Despite the fact that the SNAP-ADAR system we employed has been shown to be the most specific,^55^ all RNA base editing technologies developed thus far show abundant (if low level) off-target editing.^66^ This is especially the case in two-component systems like the one employed here. Thus, we cannot completely exclude the possibility that the output of targeted editing on MAVS (namely ISG/NF-κB activation), is at least in part the result of off-target editing. Aggregating ISG and NF-κB scores mitigates this concern as these “scores” evaluate pathway activation as a whole, thus minimizing the effect of changes in a handful of genes that display off-target editing.

## STAR★Methods

### Key resources table

**Table:** 

REAGENT or RESOURCE	SOURCE	IDENTIFIER
**Antibodies**		

Rabbit monoclonal anti-ADAR1 (clone D7E2M)	Cell Signaling Technology	Cat# 14175, RRID:AB_2722520
Rabbit monoclonal anti-GAPDH antibody, unconjugated, (clone 14C10)	Cell Signaling Technology	Cat# 2118, RRID:AB_561053
Mouse monoclonal anti-β-Actin antibody	Sigma-Aldrich	Cat# A5441, RRID:AB_476744
Calnexin antibody	Enzo Life Sciences	Cat# ADI-SPA-865-F, RRID:AB_11180747

**Bacterial and virus strains**		

DH5alpha Competent *E. Coli*	Invitrogen	Cat# 12297016
Stbl3 Competent *E. Coli*	Invitrogen	Cat# C737303

**Chemicals, peptides, and recombinant proteins**		

IFN-α	PBL Assay Science	Cat# 11100-1

**Critical commercial assays**		

NEBuilder® HiFi DNA Assembly Cloning Kit	NEB	Cat# E5520
RNeasy Plus Mini kit	Qiagen	Cat# 74134
Kapa mRNA hyperprep Kit for Illumina platforms	Kapa Biosystems, Roche	Cat# KR1352
TruSeq Stranded mRNA Library Prep Kit	Illumina	Cat# RS-122-2101
High Pure PCR template Preparation kit	Roche	Cat# 11796828001
DNase	Invitrogen	Cat# AM1907
Q5® High-Fidelity DNA Polymerase	NEB	Cat# M0491
One-step RT-PCR kit	Qiagen	Cat# 210212
CloneJET PCR Cloning Kit	Thermo Fisher	Cat# K1231
ProtoScript M-MuLV First-Strand Synthesis Kit	NEB	Cat# E6300
SsoAdvanced Universal SYBR Green Supermix	Bio-rad	Cat# 1725270

**Deposited data**		

1^st^ DLBCL cohort (n = 106), discovery cohort.	Ren et al.,Ye et al.^5^^,^^67^	CNP0001228 and CNP0001220
2^ND^ DLBCL cohort (n = 54), validation cohort 1.	Karolinska University Hospital and Uppsala University Hospitals in Sweden	PRJNA952204
3^rd^ DLBCL cohort (n = 420), validation cohort 2.	Lenz et al.^68^	GSE10846
20 FL samples, validation cohort 3.	Ren et al.^67^	
Control B cells datasets (n = 41).	Cheung et al., Li et al.^44^^,^^46^	GSE29158, GSE25840, GSE16921
Lung adenocarcinoma cohort (n = 57).	Moreno Leon^69^	GSE116959
RNAseq from all cell lines included in this study. RNAseq data SNAP experiment.	This study.	GSE145011 PRJNA963175
Proteomic data RCK8 ADAR1 wt and KO; RCK8 SNAP experiment.	This study.	PRIDE: PXD041549
**Experimental models: Cell lines**		
RCK8	DSMZ	Cat# ACC-561, RRID:CVCL_1883
A549	DSMZ	Cat# ACC-107, RRID:CVCL_0023
HEK293T	ATCC	Cat# CRL-3216, RRID:CVCL_0063

**Oligonucleotides**		

All the primers, oligos and gRNAs used in this study are listed in Table S6.	This study.	N/A

**Recombinant DNA**		

pSpCas9(BB)-2A-GFP (PX458)	Addgene^83^	Cat# 48138, RRID:Addgene_48138
lentiCRISPRv2 puro	Addgene^84^	Cat# 98290, RRID:Addgene_98290

**Software and algorithms**		

STAR software	Dobin et al.^85^	https://github.com/alexdobin/STAR
Samtools v1.12	Li et al.^91^	http://samtools.sourceforge.net/
REDItools2	Picardi et al., Picardi et al.and Pesole et al.,Lo Giudice et al.^74^^,^^75^^,^^76^	https://github.com/BioinfoUNIBA/REDItools2
MultiEditR	Kluesner et al.^89^	https://moriaritylab.shinyapps.io/multieditr/
TIMER	Li et al.^61^	https://cistrome.shinyapps.io/timer/
AEI	Roth et al.^47^	https://github.com/a2iEditing/RNAEditingIndexer
Qlucore Omics Explorer	Qlucore	https://qlucore.com

### Resource availability

#### Lead contact

Further information and requests for resources and reagents should be directed to and will be fulfilled by the lead contacts, F. Nina Papavasiliou (n.papavasiliou@dkfz.de) and Qiang Pan-Hammarström (Qiang.Pan-Hammarstrom@ki.se).

#### Material availability

Plasmids and cell lines generated in this study are available upon request.

### Experimental model and study participant details

#### Patient information and datasets

Three DLBCL cohorts were analyzed in the current study. The first cohort, the discovery cohort, consisted of 106 patients and was derived from a previously published study.^5^ The gender and the available clinical features of the patients were summarized in Table S1. The second DLBCL cohort (n = 54), validation cohort 1, were patients collected from the Karolinska University Hospital and Uppsala University Hospitals in Sweden (Table S1). The third DLBCL cohort (n = 420), as validation cohort 2, were obtained from a published dataset (GSE10846).^68^ In addition, 20 FL samples described previously,^67^ was also used as validation cohort 3 (Table S1). Informed consent was obtained from all patients, and the institutional review boards approved the study at Tianjin Medical University Cancer Hospital, Uppsala University and the Karolinska Institutet.

Data from "control B cells" were obtained from previously published datasets (GSE29158, GSE25840, GSE16921), which were derived from immobilized B cell lines (EBV transformed LCLs) from healthy individuals (n = 41).^44^^,^^46^^,^^46^ A lung adenocarcinoma cohort (n = 57) (GSE116959^69^) was also included in the study.

#### Cell lines

RC-K8 cells (DSMZ, Germany) were cultured at 37°C, 5% CO_2_, in RPMI with L-glutamine and sodium bicarbonate (Sigma), supplemented with 15% fetal bovine serum (FBS, PAN Biotech) and Penicillin/Streptomycin (Sigma). The RC-K8 cell line was established from a patient with GBC-like lymphoma, described at the time as histiocytic lymphoma (terminal, refractory stage),^70^ and later was assigned to the GCB subtype of DLBCL.^71^ However, more recent studies suggested constitutive NF-κB signaling in RC-K8 cells, a feature that is usually associated with the ABC subtype.^72^ Therefore, we performed both DNA and RNA sequencing on this cell line, and the data suggested that RC-K8 indeed be assigned to the ABC subtype.^64^ A549 cells (DKFZ, Germany) and HEK293T (ATCC) were cultured at 37°C, 5% CO_2_, in high glucose DMEM (Sigma), supplemented with 10% FBS and Penicillin/Streptomycin. All cell lines were regularly tested for mycoplasma contamination in the Multiplexion facility (DKFZ, Heidelberg, Germany).

### Methods details

#### Processing of DNA sequencing

DNA and RNA sequencing data were obtained from a previous publication.^5^ Briefly, for DNA sequencing, BWA (v0.5.9) was subsequently used to align reads to the human reference genome (hg19)^73^ and VarScan (v2.3.9) was used to detect substitutions and insertions and deletions using the defined parameters.

#### Processing of RNAseq and calling of RNA editing sites

The RNA sequencing reads were analyzed and mapped to the hg19 reference genome using SOAP2.^74^ The aligned sequencing files (BAM) were further used as input for *RED-ML,* an effective RNA editing detection method based on machine learning.^30^ The pipeline extracts candidate RNA editing sites and their corresponding features, with optimal filtering if DNA mutation information is available, then apply a logistic regression classifier to detect authentic RNA editing sites with an associated confidence score. Briefly, three classes of features were used: 1) basic read features, including the number of supporting reads of a candidate site and the putative editing frequency; 2) related to possible sequencing artifacts and misalignments, including mapping qualities of the supporting reads, the relative position of the candidate site in the mapped reads (usually 5bp away from read end as in^75^ indication of strand bias, whether the candidate site falls into simple repeat regions; 3) based on known properties of RNA editing, such as the editing type (whether it is A-to-I), whether the candidate site is in an Alu region, and its sequence context.^30^ In total, 28 features (Table S4 in ref. ^30^^)^ for every possible editing site were extracted to remove potential errors during cDNA synthesis, sequencing and mapping or contamination from genomic variants. Finally, this pipeline is concordant with more commonly used algorithms (e.g. REDITools.^74^^,^^75^^,^^76^

#### Alu editing index (AEI) and MAVS 3′UTR editing index

After removing the adapter sequence in the raw data and low-quality reads, HISAT was used to align reads to the human reference genome (hg38). The read length of DLBCL and FL samples were cut to 100 bp to avoid potential bias of read length on AEI index. AEI index in each tumor sample was then calculated using the published pipeline.^47^ MAVS 3′UTR editing index (Figure 4B) was calculated as the ratio of ‘number of reads containing an A-to-G editing’ and ‘number of total reads of each A’ in the region chr20:3848102-3855844 (hg19).

#### Calculating RNA editing at the gene level

In order to calculate the editing frequency at the gene level, all the RED-ML derived editing sites were filtered to keep only reads reporting A-to-G changes. Next, RED-ML derived edit sites were reported in less than two samples, or sites within intergenic regions were filtered out. Starting from this filtered subset of editing sites, we calculated "gene-level" editing as follows: for each gene, we first aggregated all reads covering at least one of the editing sites reported for that gene, with base quality higher than 20 and mapped quality higher than 30, and with at least 2 base pairs distance of the editing site from ends of the trimmed read (thus 7 bp from the ends of the raw read). Then the editing level of the gene was calculated as the number of reads with at least 1 edited base-pair divided by the final number of filtered reads covering that gene (Figure S1).

#### Gene set enrichment analysis (GSEA)

Gene expression levels were quantified by RSEM (RNAseq by Expectation Maximization).^79^ RSEM computed maximum likelihood abundance estimates using the ExpectationMaximization (EM) algorithm for its statistical model. The number of transcripts per million (TPM) was used to determine gene expression levels. In order to remove any possible batch effect from datasets, the expression data were log2 transformed (log2 of (TPM+1) values) and subjected to the "removeBatchEffect" command (R package Limma^80^). The normalized expression values were analyzed by Qlucore Omics Explorer (Qlucore AB, Lund, Sweden) or GSEA (Broad Institute, Cambridge, USA).

#### Proteomics analysis in DLBCL samples

Fourteen DLBCL samples were used for mass spectrometry-based proteomics analysis. The TMT10 isobaric labeling was applied in two batches; each contained nine samples and one pooled control. According to the manufacturer’s instructions, proteins were extracted by Trizol reagent (Invitrogen, Paisley, UK). Precipitated proteins dissolved in urea 8M buffer, and the concentration was measured by BCA assay. 100 μg protein per sample was reduced and alkylated by DTT and IAA respectively to a final concentration of 10 mM. Then, samples were digested using LysC (Wako) and followed with trypsin (Promega, Madison, WI). Acetonitrile was added to a final volume of 10%, and TMT was added at a ratio of 2.5:1. TMT labeled samples were then combined and purified. Each pooled sample was fractionated into eight fractions by high pH reversed-phase liquid chromatography, and then all fractions were analyzed with an Orbitrap Fusion mass spectrometer (Thermo, San Jose, CA).

#### Proteomics analysis in cell lines and in the site-directed RNA editing experiment (shown in Figure 4)

Sample preparation:

Proteins were loaded on SDS-PAGE-gel, which ran only a short distance of 0.5 cm. After Commassie staining the total sample was cut out unfractionated and used for subsequent Trypsin digestion according to a slightly modified protocol described by Shevchenko et al. (Shevchenko, Andrej, et al. "In-gel digestion for mass spectrometric characterization of proteins and proteomes."*Nature protocols* 1.6 (2006): 2856.) on a DigestPro MSi robotic system (INTAVIS Bioanalytical Instruments AG).

LC-MS/MS analysis – DIA:

Dried peptides were resuspended in reconstitution buffer (2.5% 1,1,1,3,3,3-Hexafluoro-2-propanol, 0.1% TFA in water) prior to analysis. A 120 min LC-MS/MS analysis was carried out on an Ultimate 3000 UPLC system (Thermo Fisher Scientific) directly connected to an Orbitrap Exploris 480 mass spectrometer. Peptides were online desalted on a trapping cartridge (Acclaim PepMap300C18, 5 μm, 300 Å wide pore; Thermo Fisher Scientific) for 3 min using 30 μl/min flow of 0.05% TFA in water. The analytical multistep gradient (300 nL/min) was carried out on a nanoEase MZ Peptide analytical column (300 Å, 1.7 μm, 75 μm × 200 mm, Waters) using solvent A (0.1% formc acid in water) and solvent B (0.1% formic acid in acetonitrile). For 102 min the concentration of B was linearly ramped from 4% to 30%, followed by a quick ramp to 78%, after 2 min the concentration of B was lowered to 2% and a 10 min equilibration step appended. Eluting peptides were analyzed in the mass spectrometer using data independent acquisition (DIA) mode. A full scan at 120k resolution (380–1400 *m*/*z*, 300% AGC target, 45 ms maxIT. profile mode) was followed by 47 windows of variable isolation width^81^ (400–1000 *m*/*z*, 1000% AGC target, 30k resolution, 54 ms maxIT, centroid) for fragment spectra acquisition. Normalized CE was set at 28%.

Data analysis: DIA files were processed in Spectronaut (16.2.220903.53000) via directDIA with default settings adapted as follow: In the Pulsar Search, Result Filters were set to Max 1800 *m*/*z* and Min 300 *m*/*z* and the relative Intensity was set to a min 5%. In the DIA Analysis, Quantification was set to MaxLFQ. A human UniProt database (from 2021 to 09-29) containing 79,038 entries was used for the database search. The mass spectrometry proteomics data have been deposited to the ProteomeXchange Consortium via the PRIDE^82^ partner repository with the dataset identifier PXD041549.

#### Calculation of the interferon-stimulated gene (ISG), NF-κB and T cell exhaustion scores

Thirty-eight genes representing ISG signatures described previously^28^ were used to calculate the ISG score. Two hundred seventy-nine genes overlapping from our expression dataset and reported as NF-κB downstream genes from Boston University’s gene resources [κhttps://www.bu.edu/NF-κB/gene-resources/target-genes/] were used for calculating the NF-κB score. T cell exhaustion score was calculated similarly (from the genes in Figure 5). For each ISG or NF-κB sets, scores were calculated based on the methods as in the original publication.^28^

#### Establishment of ADAR1 knockout RC-K8 cell line

The knockout of ADAR1 in RC-K8 cells was performed by using pSpCas9(BB)-2A-GFP (PX458, Addgene plasmid #48138), which was a gift from Feng Zhang.^83^ DNA oligos #7, #8 and #9 (Table S6) were cloned into this plasmid linearized by restriction digestion (BbsI) using NEBuilder HiFi DNA Assembly Cloning Kit (NEB). We, therefore, obtained two plasmids for targeting respectively exon 3 (sgRNA 1, designed using eCRISP (www.e-crisp.org)) and exon 4 (sgRNA 2, from^21^ of *ADAR1* and one plasmid as a non-targeting control (NT-ctrl) (Figure S9A). RC-K8 cells were then electroporated using AMAXA Nucleofector 2b device (Lonza). Briefly, 2 x 10^6^ cells were resuspended in 100 μL of V Nucleofector Solution, and 2 μg of plasmids were used for transfection following the Nucleofector Program L-013. 48 h after transfection, GFP positive cells were sorted and plated (one cell per well) in 96-well plates. The clonality was validated by visual inspection with a microscope, and positive clones were screened by Sanger sequencing.

Knockout of ADAR1 was validated by Western blot (cat. no.#14175, Cell Signaling Technology) with GAPDH as a control (cat. no.#2118, Cell Signaling Technology). Three potential knockout clones (clones 3, 14 and 18) were obtained after screening around 250 clones (Figure S9B). These clones were also validated by amplifying *ADAR1* targeted region from genomic DNA (using oligos #10 and #11) and RNA (using oligos #44 and #45) followed by either Sanger sequencing or cloning (#K1231, ThermoFisher) followed by Sanger sequencing. In addition, RC-K8 control (Ctrl) cells were obtained from three different clones from RC-K8 cells transfected with the control plasmid. Finally, for experiments in which we wanted to avoid clonal effects (e.g., Figures S11B, S12A and D) we sorted a GFP positive polyclonal population of RC-K8 cells transfected with the NT-ctrl plasmid.

#### Establishment of ADAR1 knockout A549 cell line

The knockout of ADAR1 in A549 cells was performed using LentiCRISPRv2 (#52961, Addgene), which was a gift from Feng Zhang.^84^ DNA oligos #1–2 and #3–4 (Table S6) were cloned into this vector for the knockout of ADAR1. As a non-targeting control, lentiCRISPR-NT (Lenti-NT) was cloned using oligos #5 and #6 based on non-targeting control 800 from the GeCKO v2 library.^84^ Positive clones were screened by Sanger sequencing.

These plasmids, in combination with pCMV-DR8.91 (coding for HIV gag-pol) and pMD2.G (encoding the VSV-G glycoprotein) (kind gifts from Prof. Didier Trono, Lausanne, Switzerland), were calcium-phosphate transfected into HEK293T cells for lentiviral particle production (ratio 3:1:3). After 48–72 h, cell-free supernatant was collected and used for transduction of A549 cells. The transduced cells were selected with puromycin (1 μg/mL). After approximately two days (as soon as non-transduced cells died), the ADAR1 knockout pools were seeded in 96-well plates in a limiting dilution (0.5 cells/well); Upon expansion of single clones, knockout of ADAR1 was validated by Western blot after IFN-α stimulation, using β-Actin (#A5441, Sigma-Aldrich) or Calnexin (#ADI-SPA-865-F, Enzo Life science) as controls. Lenti-NT control cells were kept polyclonal. After screening, two clones (5 and 7) were obtained with completely abolished ADAR1 (p110 and p150) expression (Figure S9G).

#### RNA-seq data and processing for A549 and RC-K8 (ADAR1 WT and KO)

RNA was extracted using RNeasy mini kit (Qiagen), and RNA-seq libraries were prepared from A549 Lenti-NT control and ADAR1 KO clone 5, and from RC-K8 ctrl clone 2 and ADAR1 KO clone 14 (Figure S8). For experiments related to A549, RNA was processed with Kapa mRNA hyperprep Kit for Illumina platforms (Kapa Biosystems, Roche) and KAPA Single-Indexed Adapter Kit for Illumina Platforms (Kapa Biosystems, Roche). For experiments related to RC-K8, libraries were prepared using the TruSeq Stranded mRNA Library Prep Kit from Illumina. Libraries were sequenced with the Illumina HiSeq 2000 v4 or with NovaSeq 6K PE 100 S4 technology. After removing the adapter, the reads were mapped to the human reference genome hg19 with the STAR software.^85^ Aligned RNA-seq were sorted and indexed with samtools v1.12. Picard (MarkDuplicates function) was used for removing potential PCR duplicates. RefGene from UCSC was employed for gene annotation. Transcript quantifications were perfomed using Salmon (version 0.14.2) (CIT), TMM values were computed using “house-script” and EdgeR(ver) package.^86^^,^^87^ Single Nucleotide Variations (SNVs) were called by REDItools2.^74^^,^^75^^,^^76^ For the variant calling between the ADAR WT and KOs, all positions must be well-covered (min. 50 reads per replicate) in both groups, and RNA editing site events were considered for those that showed at least 0.2 as editing frequency in WT. Edited positions were annotated using Ensembl Variant Effect Predictor^88^ (VEP version 107.0).

#### Editing-site validation

Genomic DNA (gDNA) and RNA were prepared from RC-K8 cells, including ADAR1 WT and KO. gDNA was extracted using the High Pure PCR template Preparation kit (Roche) following manufacturer instructions. PCR amplification was then performed using Q5 High-Fidelity DNA Polymerase (NEB). RNA was extracted using the RNeasy Mini kit (Qiagen) and treated with DNase (Invitrogen). A one-step RT-PCR kit (#210212, QIAGEN) was used. The PCR products were purified (#740609, Macherey-Nagel) and analyzed by Sanger sequencing (Microsynth AG, Switzerland). Quantification of editing was performed directly from the chromatogram using MultiEditR.^89^ The editing sites identified in DLBCL patients within *CARD11* and *TP73* transcripts by RED-ML were validated by amplifying the targeted region from both genomic DNA and RNA followed by cloning (#K1231, ThermoFisher) and Sanger sequencing.

#### Interferon-alpha (IFNα) stimulation

A549, RC-K8 or HEK293T were seeded at different cell densities (10,^5^ 5x10^5^, 2.5x10^5^ cells/ml, respectively) in 12 well plates, in a total volume of 1 mLmedia containing 200 U/ml of IFN-α (#11100-1, pbl assay science). After 16 h, cells were collected, and RNA was extracted using a Qiagen RNAeasy plus kit and treated with DNase (Invitrogen, Cat# AM1907). cDNA synthesis was performed using ProtoScript M-MuLV First-Strand Synthesis Kit (NEB) using oligo-dT and 1 μg of total RNA DNAse digested as input. Two microliters of a 1:2 diluted cDNA were used to set up a 10 μL qPCR reaction using SsoAdvanced Universal SYBR Green Supermix (Bio-rad, Cat# 1725270). qPCR was then performed on two genes known to be upregulated after IFN stimulation (*RIG-I, ISG15*) and *MAVS*. All experiments were performed in triplicates and all the primers used are listed in Table S6.

### Site-directed RNA editing restoration

RC-K8 *ADAR1*^−/−^ cell line (clone 18) were transduced with lentiviral particles containing the pLentipuroplasmid (Addgene) carrying SNAP-ADAR1 [E406Q] fused to GFP, produced by the Stafforst lab using the virapower expression system and following the protocol provided by the manufacturer (ThermoFisher). For transduction, 3x10^5^ cells were seeded in a 24-well with 300 μL of RPMI medium with 2% FBS, and viral particles were added at a MOI of 0.5. After 24 h, 200 μL of RPMI medium with 10% FBS was added. 6–7 days after transduction, positively transduced cells were selected using FACS via the GFP marker expressed with SNAP-ADAR1.

BG-linked guides were purchased from Biospring and BG-lated by the Stafforst lab.^90^ Then, 200 pmol each of gRNA MAVS BB-455 and BB-456, or a non-BG gRNA CTRL (#40–43, Table S6), were directly electroporated into RC-K8 ADAR1^−/−^ SNAP-ADAR1[E406Q]-GFP, using AMAXA Nucleofector 2b device (Lonza) following a specific protocol for this experiment. Briefly, 1 x 10^6^ cells per sample were resuspended in 100 μL of V Nucleofector Solution and gRNAs were transfected into the cells using the Nucleofector Program X-001.

RNA was extracted 24 h after transfection using the RNeasy Mini kit (Qiagen) for *MAVS* RNA editing quantification and qRT-PCR analysis. The same RNA was also used to prepare RNA-seq libraries using the TruSeq Stranded mRNA Library Prep Kit from Illumina. Libraries were sequenced with the Illumina HiSeq 4000 technology, generating 100-nucleotide, paired-end reads. RNA-seq data were processed as described above (paragraph "RNA-seq data and processing for A549 and RC-K8 (ADAR1 WT and KO)"). Due to the low coverage on *MAVS* (Figure 4C), we pooled data from the two replicates for each sample (gMAVS or gCTRL) for the variant calling. All positions must be decently covered (min. 10 reads per pull) in both groups, and RNA editing site candidates were considered those that showed at least 1 read carrying an A-to-G variation.

#### Prediction of tumour-infiltrating immune cells in DLBCL

Tumor infiltration of different immune cell types (CD4^+^T cells, CD8^+^T cells, neutrophils, macrophages and dendritic cells) were predicted using the online tool TIMER (Tumor IMmune Estimation Resource; https://cistrome.shinyapps.io/timer/).^61^

### Quantification and statistical analysis

#### Quantification and statistical analysis in this article were performed in R or prism (GraphPad)

All of the statistical details for each experiment can be found in the figure legends. The p values in Figures 1F and 1G, 5A, 5C-F5H-5J were calculated using Mann-Whitney U test. The p value in Figure 2A was calculated using Fisher’s exact test. The p and r values in Figures 1B, 1E, and Figure 3 were calculated using Pearson correlation coefficient.

## Data Availability

• RNAseq from all cell lines included in this study has been deposited in GEO under accession numbers GSE145011 and PRJNA963175. In addition, WGS and RNASeq data of DLBCL patients have been deposited in the China National GeneBank Sequence Archive of China National GeneBank Database with accession nos. CNP0001228 and CNP0001220.^67^ Finally, proteomic data from RCK8 ADAR1 WT and KO and from the SNAP experiment (Figure 4) have been deposited in PRIDE with accession number PXD041549. • This paper does not report original code. • Any additional information required to reanalyze the data reported in this paper is available from the lead contact upon request.

## Supplemental information

Document S1. Figures S1–S14 and Tables S2, S4–S6

Table S1. Patients information, related to Figures 1 and 3

Table S3. RNA editing and DNA mutations in DLBCL samples (A) Unique editing sites in more than 10% of DLBCL (filtering against editing sites identified in normal B cells). (B) Results of Wilcoxon rank-sum tests for differences of mutations number per gene between groups (related to Figure 2)

Data S1. Results from differentially edited editing sites analysis in gMAVS samples in comparison to gCTRL samples, related to Figure 4D
