# Supplementary material for: ADAR1-mediated RNA editing promotes B cell lymphomagenesis
Source: iScience. 2023 May 12;26(6):106864. doi: 10.1016/j.isci.2023.106864 (PMC10225930; doi:10.1016/j.isci.2023.106864)
Supplement: Document S1. Figures S1–S14 and Tables S2, S4–S6 [file mmc1.pdf]

iScience, Volume *26*

Supplemental information

ADAR1-mediated RNA editing

promotes B cell lymphomagenesis

Riccardo Pecori, Weicheng Ren, Mohammad Pirmoradian, Xianhuo Wang, Dongbing Liu, Mattias Berglund, Wei Li, Rafail Nikolaos Tasakis, Salvatore Di Giorgio, Xiaofei Ye, Xiaobo Li, Annette Arnold, Sandra Wüst, Martin Schneider, Karthika-Devi Selvasaravanan, Yvonne Fuell, Thorsten Stafforst, Rose-Marie Amini, Kristina Sonnevi, Gunilla Enblad, Birgitta Sander, Björn Engelbrekt Wahlin, Kui Wu, Huilai Zhang, Dominic Helm, Marco Binder, F. Nina Papavasiliou, and Qiang Pan-Hammarström

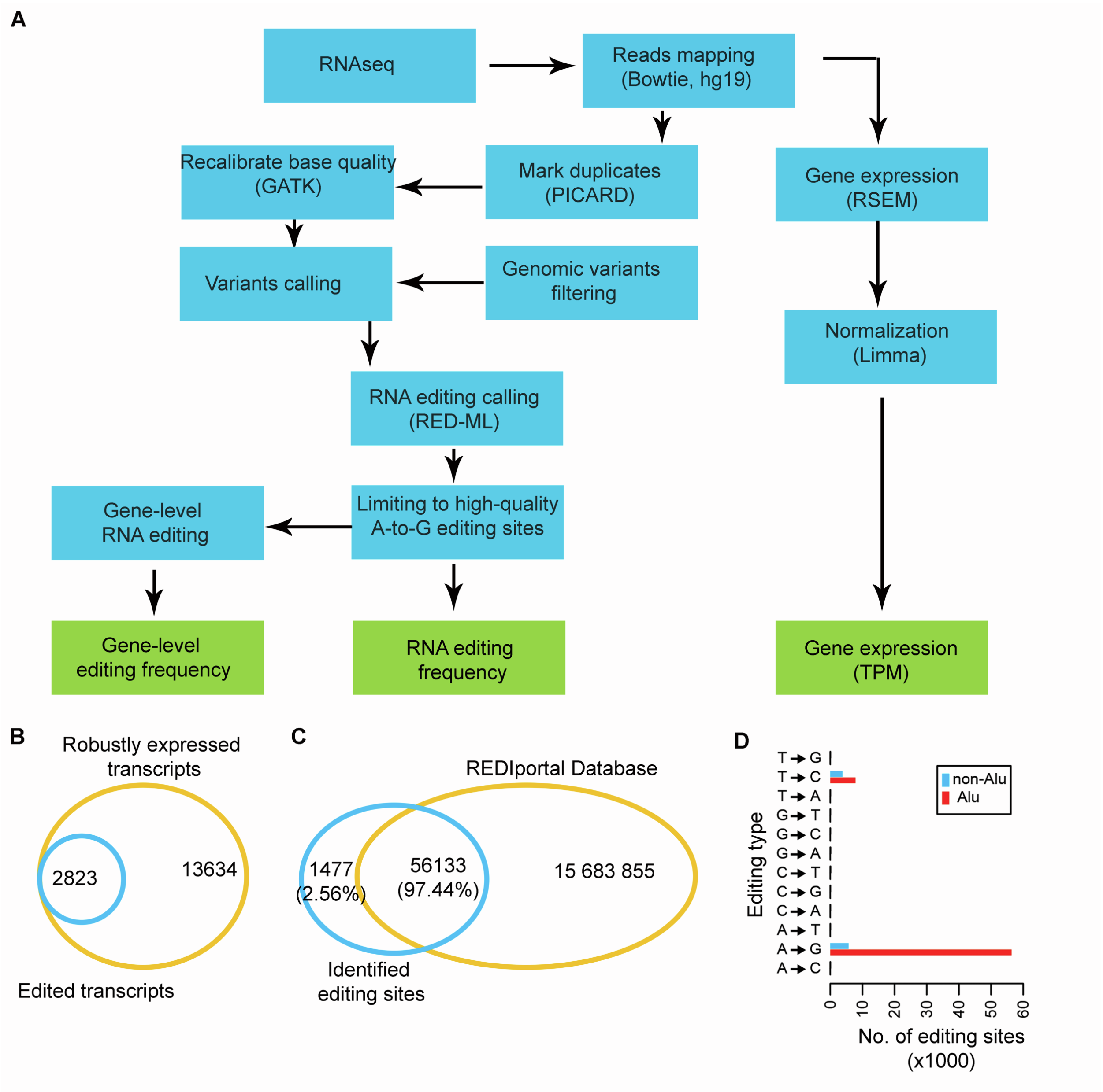

Figure S1. **The workflow to characterize RNA editing events in DLBCLs (Related to Figure 1).** (**A**) The general pipeline (RED-ML) of the data analysis for identification and quantification of RNA editing positions and the editing level and the RNA expression. **(B)** The Venn diagram shows the total number of transcripts detected and the number of transcripts edited in our DLBCL cohort. **(C)** Venn diagram presents the total identified RNA editing sites in this study and their overlap with the REDIportal database. **(D)** The editing type of RNA editing sites identified by RED-ML. The most abundant editing type was A-to-I (A-to-G) editing (represented as T-to-C for genes transcribed from the bottom strand).

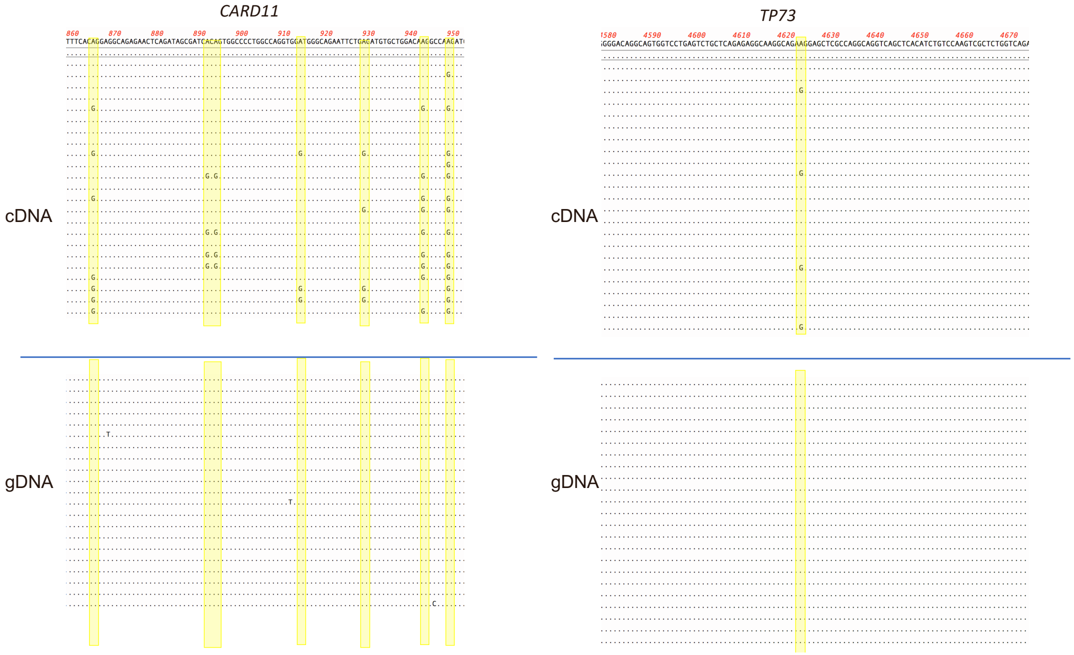

Figure S2. Validation of editing sites identified by RED-ML (Related to Figure 1). Sequences were aligned following targeted PCR amplification of cDNA and gDNA, cloning, and Sanger sequencing of bacterial colonies for *CARD11* (left) and *TP73* (right) transcripts. Editing sites identified by RED-ML are highlighted in yellow. The lack of base change in the gDNA indicates that the A-to-G transitions identified by RED-ML are actual RNA editing events.

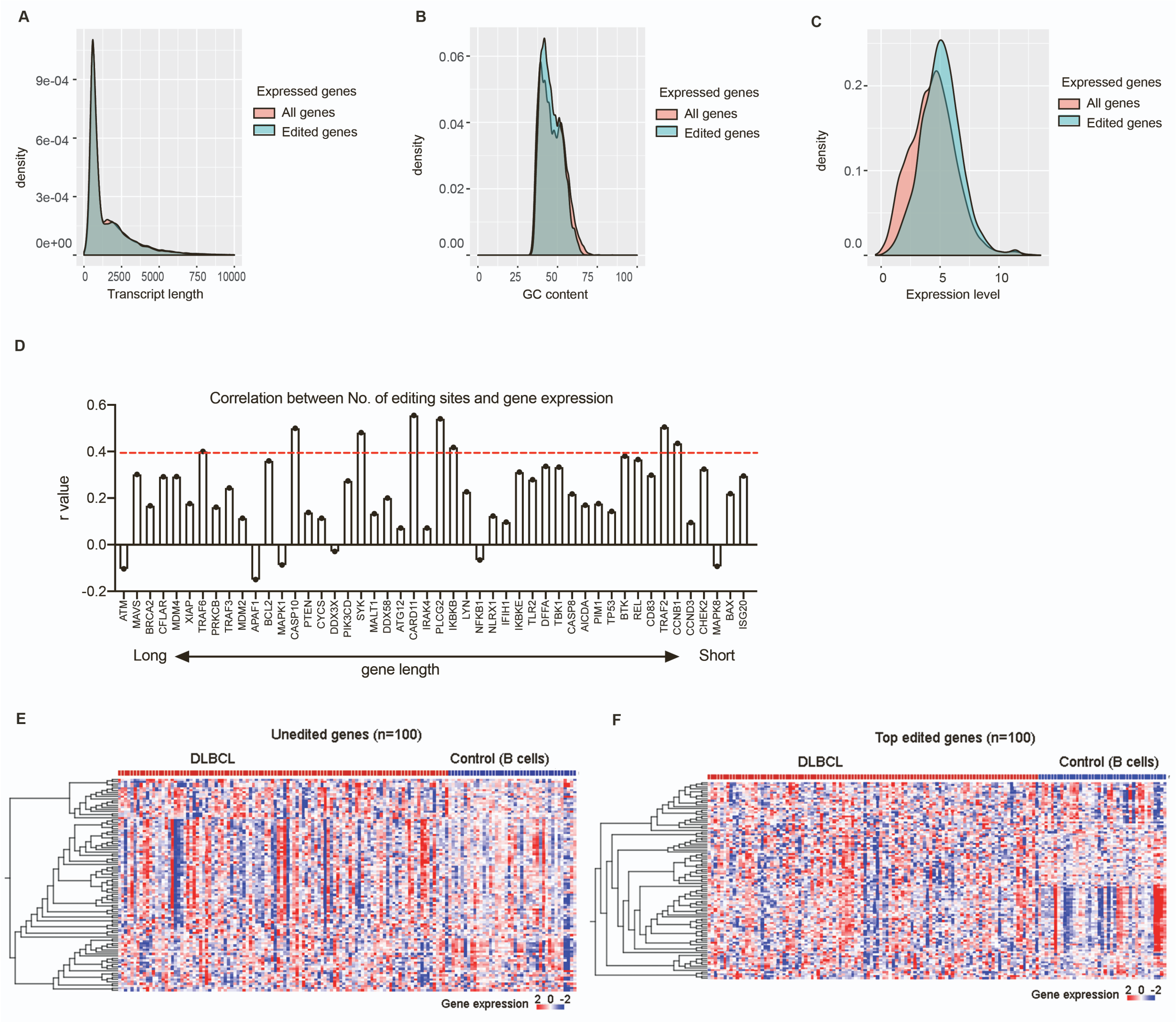

Figure S3. Features of *ADAR1* expression and RNA editing identified in DLBCL (Related to Figure 1). Overlapping analyses between edited transcripts (in red) and all detected transcripts (in green) demonstrate that editing is not affected by (**A**) transcript length, (**B**) GC content, or (**C**) global gene expression level. **(D)** Additionally, focusing more locally on the edited transcripts, we do not find a correlation between the number of edited sites and gene expression levels (an r value higher than 0.4 indicates a strong correlation). Pearson correlation coefficient was used for r values. **(E-F)** Finally, we selected 100 expressed but unedited transcripts and compared them to the top 100 edited transcripts in control B cells and within DLBCL samples. We did not find significant differences in mRNA expression of edited transcripts between B cells and DLBCL. Therefore, the increase in editing in DLBCL is not explained by the increase in gene expression (and consequent increase in editing calling).

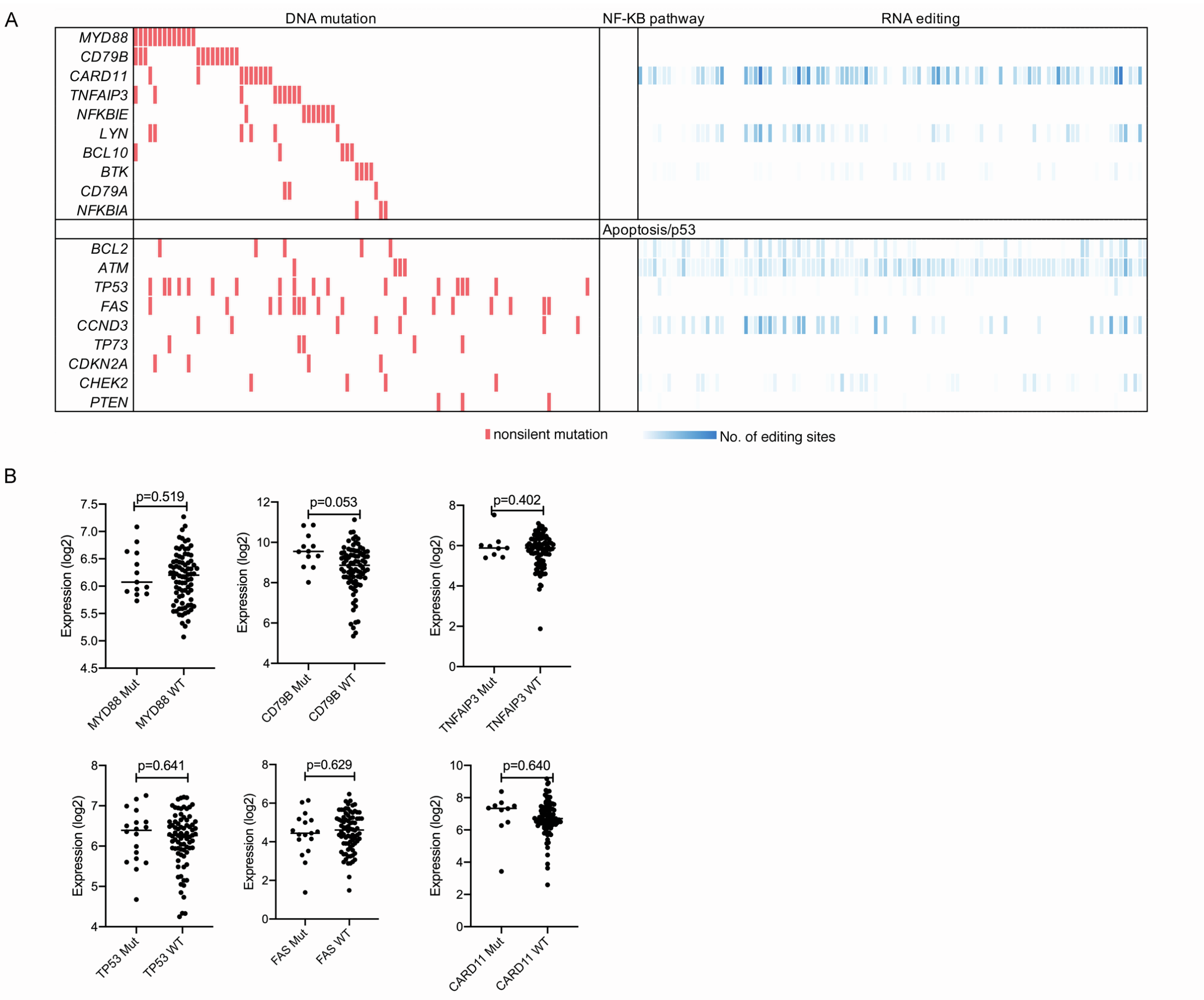

Figure S4. Comparison of gene expression, RNA editing, and DNA mutation in the indicated pathways (Related to Figure 2). (**A**) Inverse correlation between DNA mutation and RNA editing (complementing Figure 2). Mutated genes (mutation frequency more than 3%) were assigned to a KEGG pathway and then assessed for editing. We identified NF-κB (FDR $q<0.001$) and TP53/Apoptosis (FDR $q<0.001$) as significantly mutated pathways in our cohort, but not the RIG-I pathway. Similar to Figures 2B and 2D, some genes were frequently mutated but not edited, such as MYD88, CD79B, TNFAIP3 etc. (regardless of their gene expression status). Therefore, there is an inverse correlation between mutation and editing as between editing and mutation (**B).** Mutated genes whose transcripts do not show RNA editing are not differentially expressed between patients, regardless of their mutation status. A sampling of such genes is shown. Data was represented as mean +/- SD and p values were calculated using the Mann-Whitney U test.

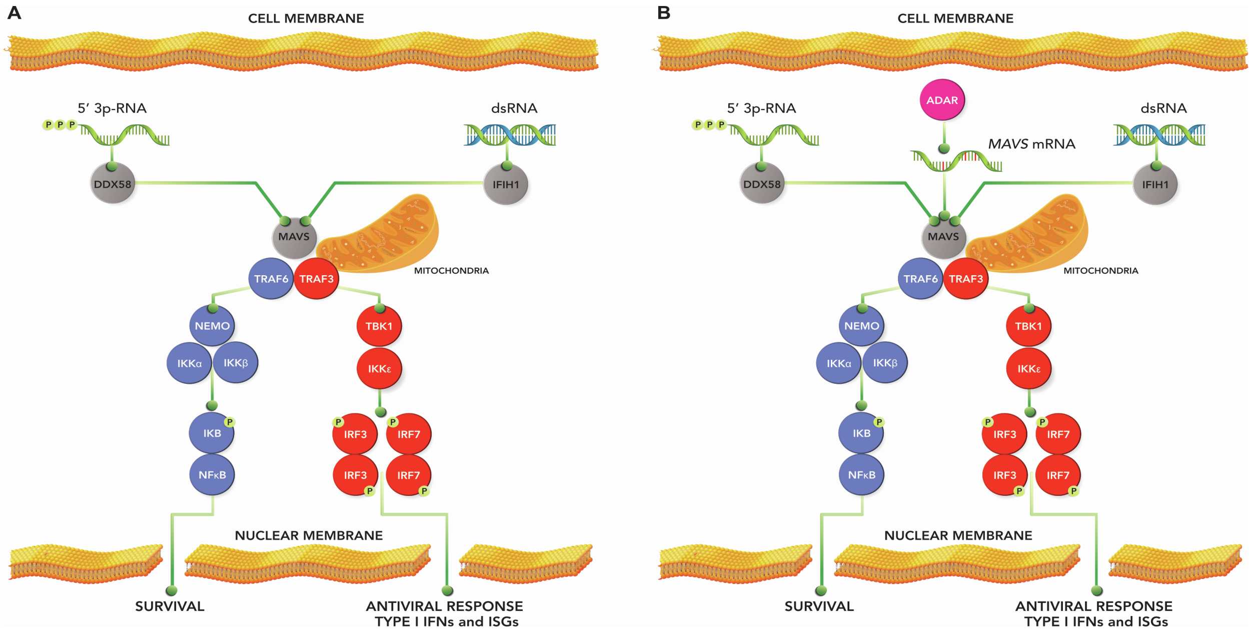

Figure S5. The central role of MAVS in the RLR pathway signaling (Related to Figure 2-4). (A) Cartoon of the RLR signaling pathway. A viral infection drives the pathway via DDX58 or IFIH1, and MAVS has the central role as an adaptor for the downstream signaling outcomes, namely, the antiviral response through TBK1/IKKε engagement (in red) and survival through IKKα/β/NF-κB signaling (in blue). (**B**) Cartoon showing *MAVS* 3’UTR ADAR1-mediated editing as a new regulation mechanism for ISG and NF-κB pathways. In the proposed model, the overexpressed ADAR1 induces RNA editing within *MAVS* 3’UTR, leading to an increase of MAVS and following activation of ISG and NF-κB pathways.

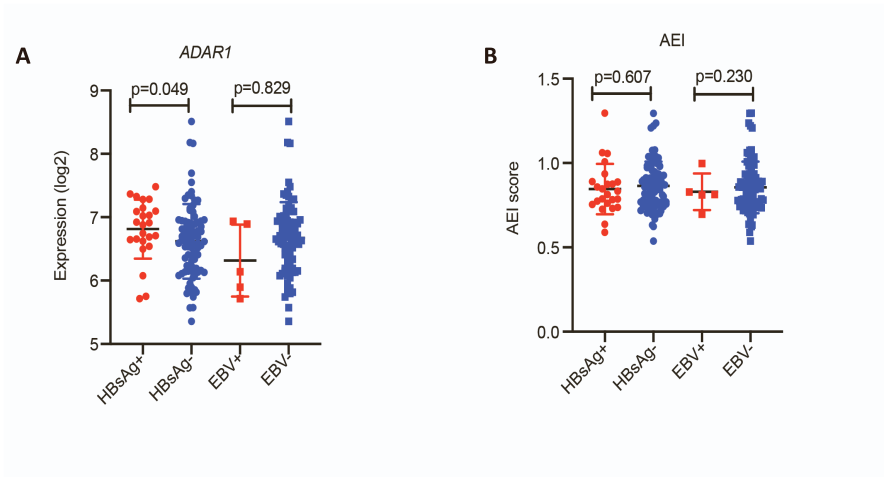

Figure S6. The association of viral infection and *ADAR1* expression and RNA editing levels in DLBCLs (Related to Figure 3). (**A**) *ADAR1* expression is slightly higher in the HBV^+ of DLBCL samples but not in the EBV^+ DLBCL samples. (**B**) RNA editing, measured as AEI, is affected by neither HBV nor EBV infection status. Data was represented as mean +/- SD and p values were calculated using the Mann-Whitney U test.

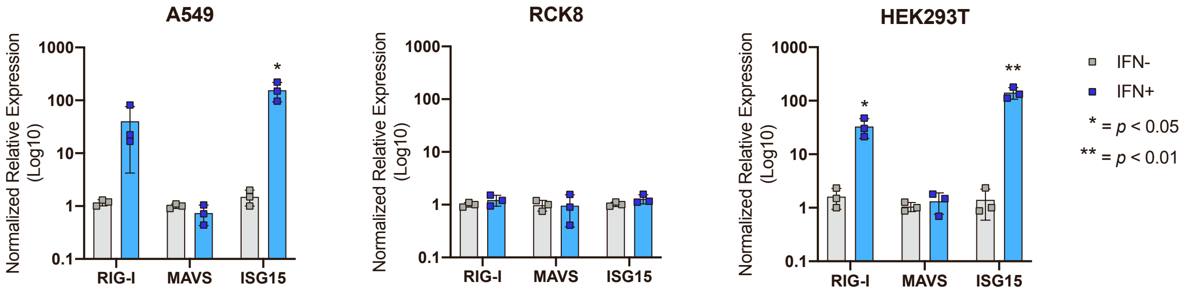

Figure S7. *MAVS* is not an interferon-stimulated gene (Related to Figure 3). The bar plots represent relative mRNA expression measured by qPCR for three cell lines following IFNα stimulation. The expression was normalized on samples that were not treated with IFNα. *PGK1* was always used as a housekeeping gene. A two-tailed t-test was used to compare the differences. *, p<0.05; **, p<0.01. Data are represented as mean +/- SD.

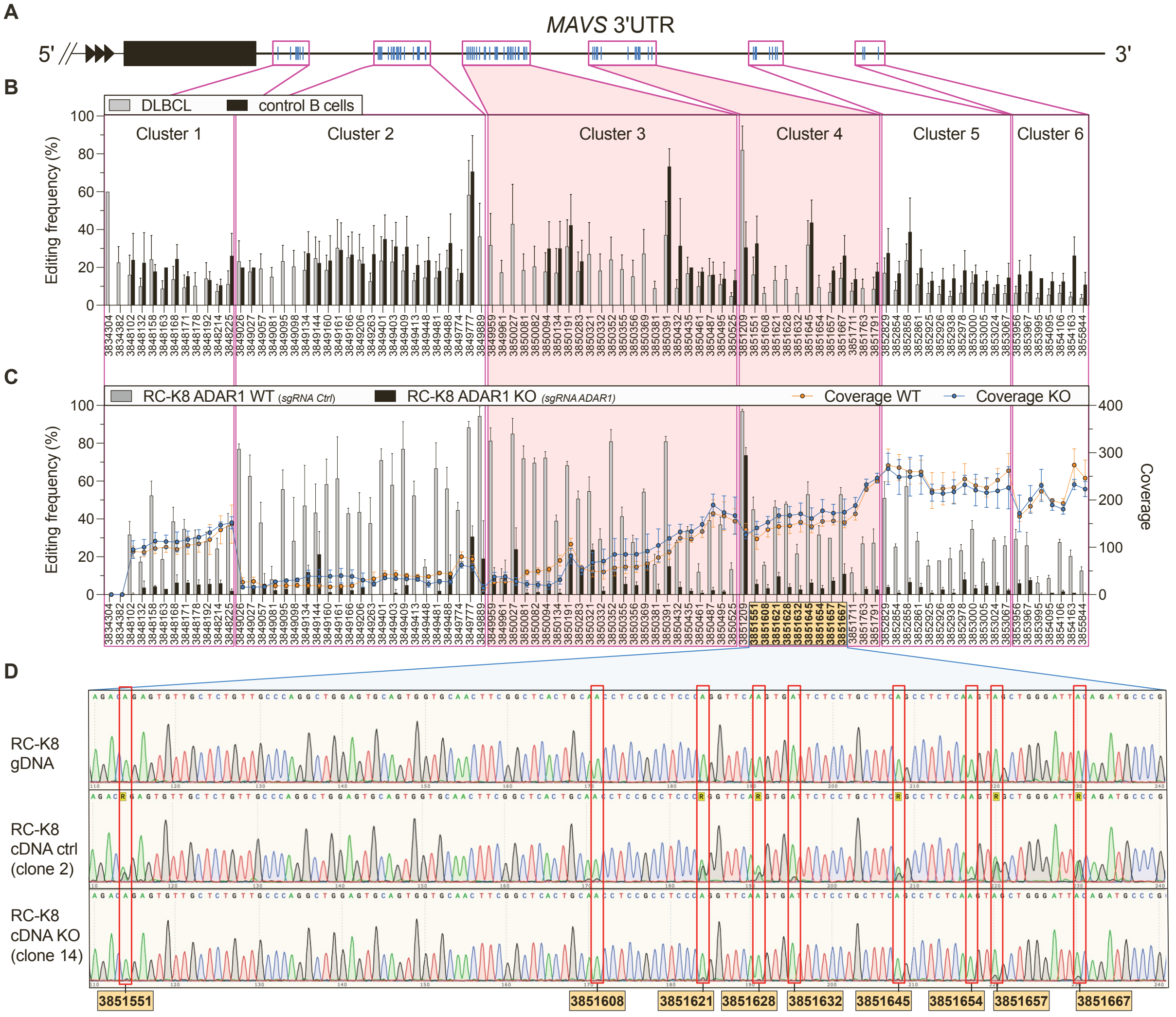

Figure S8. RNA editing clusters within *MAVS* 3'UTR in DLBCL and control B cells (Related to Figure 4). (**A**) Representation of A-to-G RNA editing sites (blues sticks) within *MAVS* 3'UTR in DLBCL. (**B**) These editing sites are organized into six clusters, corresponding to all the editing sites detected in *MAVS* within our DLBCL cohort. The bar graph shows RNA editing frequency at each site identified from DLBCL (grey bars) and B cells from healthy individuals (black bars), allowing the visualization of differently edited regions between the two groups (mainly clusters 3 and 4). (**C**) Editing frequency of each site identified from RC-K8 ADAR1 WT (Ctrl clone 2) and KO (clone 14) (grey and black bars, respectively). (**D**) Sanger sequencing validation of RNA editing sites from cluster 4. Sequencing results are shown for the gDNA of WT RC-K8, cDNA of RC-K8 Ctrl (clone 2), and RC-K8 ADAR1 KO (clone 14). All the highlighted positions (within red rectangles) were validated ADAR1 RNA editing sites due to their absence in gDNA, presence in cDNA of RC-K8 Ctrl, and their disappearance or substantial reduction in the cDNA of ADAR1 ko clone. R = purine (A or G).

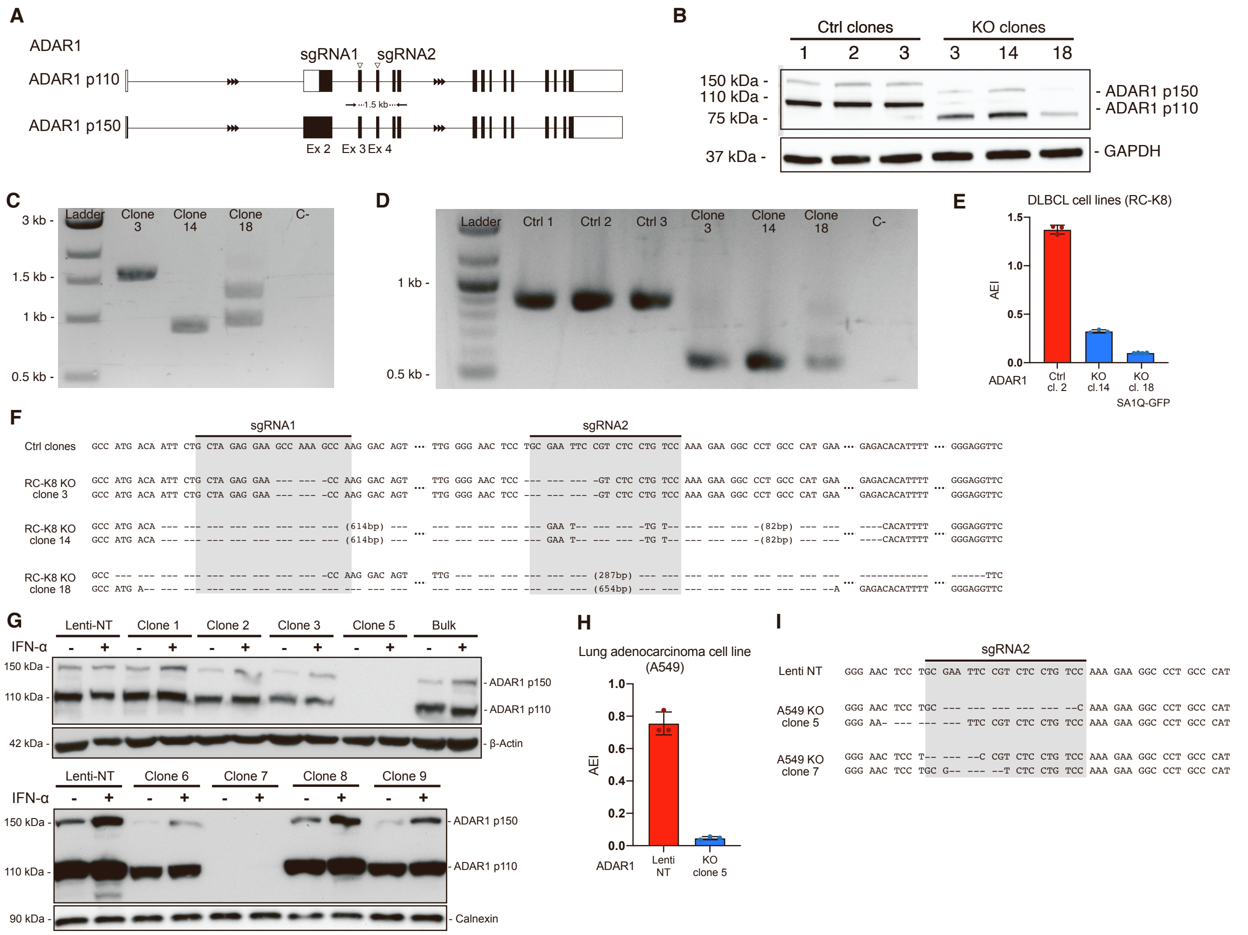

Figure S9. Characterization of RC-K8 and A549 ADAR1 knockout clones (Related to Figure 4). (**A**) Diagram of the human *ADAR1* gene, with the two isoforms p110 and p150, the sites targeted by sgRNAs, and the primers used for PCR in (C). (**B**) Western blot shows the expression of p110 and p150 in RC-K8 ADAR1 wild type (ctrl) and knockout (ko) clones (3, 14, and 18). These three ADAR1 KO clones show two shifted bands corresponding to deletion mutants of ADAR1 p110 and p150. (**C**) Agarose gel of PCR amplicons was obtained from the gDNA of ADAR1 KO clones 3, 14, and 18 (using oligos #10 and #11). As shown in (A), the expected size of the amplicon is 1570bp. These bands were used to define the specific genotype for each ADAR1 KO clone. (**F**). (**D**) Agarose gel of RT-PCR amplicons of *ADAR1* (using oligos #44 and #45). All KO clones showed a smaller amplicon size compared to the control clones. (**E**) The bar plot shows the total RNA editing level, measured as AEI for the control clone 2 and the knockout clones 14 and 18; clone 18 stably expresses SNAP-ADAR1[E406Q]-GFP (SA1Q-GFP). AEI was calculated on RNA-seq data generated in triplicates or quadruplicates for each sample. (**F**) Genotypes RC-K8 KO clones are shown. (**G**) Western blot of A549 KO clones and bulk with or without IFN-α stimulation. (**H**) The bar plot shows the total RNA editing level, measured as AEI for the Lenti-NT population control and the knockout clone number 5. AEI was calculated on RNA-seq data generated in triplicates for each sample. (**I**) Genotypes of A549 KO clones 5 and 7. For panels E and H, data are represented as mean +/- SD.

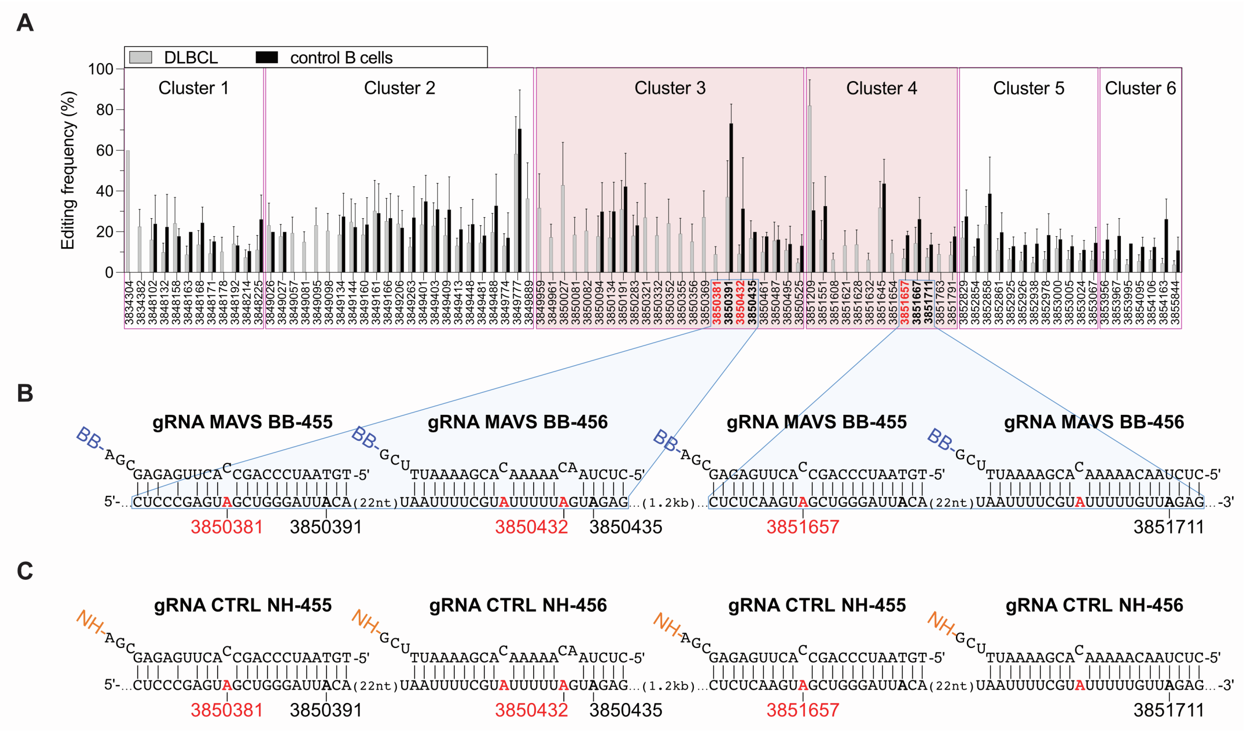

Figure S10. Rational design of gRNA for SNAP-ADAR targeting system (Related to Figure 4). (**A**) Bar graph showing RNA editing frequency at each editing site for DLBCL (grey bars) and B cells from healthy individuals (black bars), allowing the visualization of differently edited regions between the two groups (mainly clusters 3 and 4). (**B**) Two gRNAs (BB-455 and BB-456) were designed to recruit SA1Q-GFP simultaneously at two different locations in clusters 3 and 4 (highlighted in light blue), aiming to induce RNA editing in several locations within the *MAVS* 3'UTR (all the red A). (**C**) The same gRNAs without the BB group (NH-455 and NH-456) cannot recruit SA1Q-GFP and were used as control. BB = BisBg group.

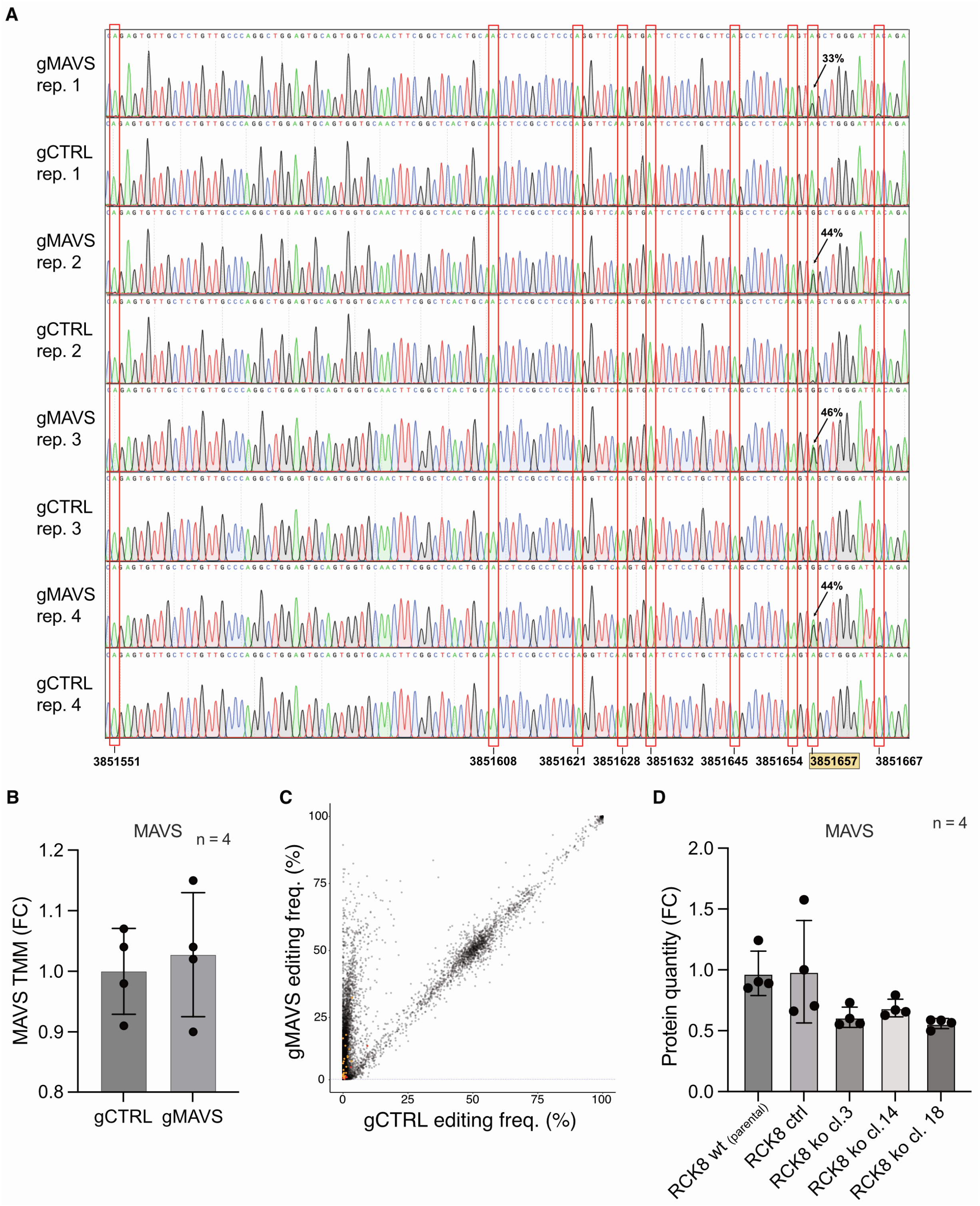

Figure S11. The re-introduction of A-to-G editing within *MAVS* 3'UTR modulates the inflammatory output of the NF-κB and type-I IFN signaling cascades (Related to Figure 4). (**A**) Sanger traces for the results shown in Figure 4. Quantification of editing was performed directly from Sanger traces using MultiEditR[1]. (**B**) NGS read counts were used for calculating TMM[2] and thus quantify mRNA levels of

MAVS after editing restoration (vs. control). (**C**) Scatterplot representing off-target analysis transcriptome-wide for gMAVS and gCTRL samples. Each dot represents one editing site. In yellow are represented all the MAVS significantly edited sites, and in red are the other MAVS editing sites. (**D**) MAVS protein quantification via mass spectrometry in the RC-K8 ADAR1 wt, ctrl, and KO. For panels B and D, data are represented as mean +/- SD.

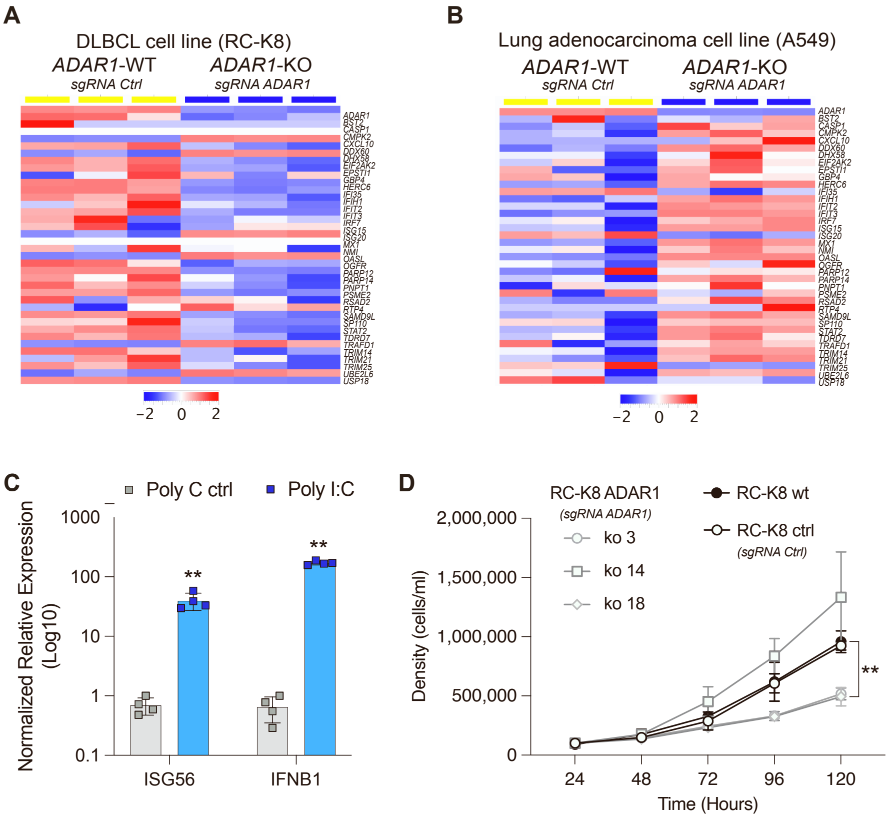

Figure S12. The analysis of ISG score in the absence of *ADAR1* in different cell lines (Related to Figure 4). (A) The heatmap shows the expression of ISG in *ADAR1*-WT and *ADAR1*-KO RC-K8 cells. **(B)** The heatmap shows the expression of ISG in *ADAR1*-WT and *ADAR1*-KO A549 cells. **(C)** RC-K8 cells are not deficient in their ability to respond to poly I:C. The bar plot represents mRNA relative expression measured by qPCR on RC-K8 following Poly I:C or Poly C control stimulation. The expression is normalized on the control sample. *PGK1* was used as housekeeping gene. A two-tailed t-test was used to compare the values of the treated samples versus the non-treated samples. A p-value of 0.01 (**) was considered statistically significant. (**D**) RC-K8 cells grow at a slower rate in the absence of ADAR1. A two-tailed t-test was used to compare the values at 120h. A p-value of 0.01 (**) was considered statistically significant. For panels **C** and **D,** data are represented as mean +/- SD.

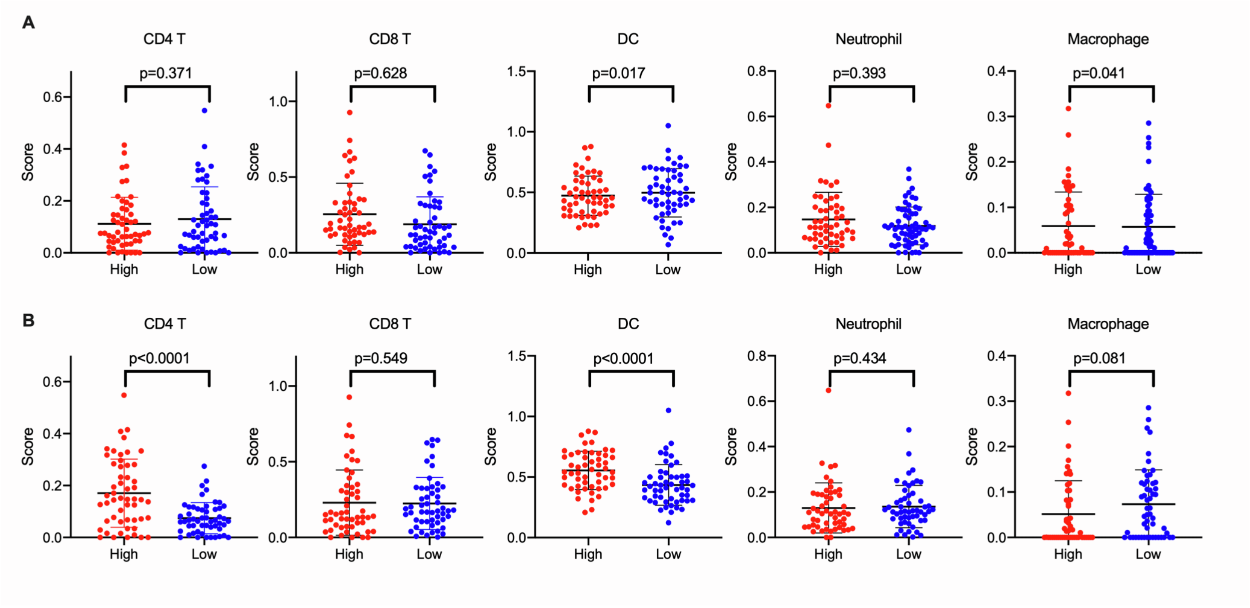

Figure S13. *MAVS* and *ADAR1* expression within DLBCL tumor cells is associated with T-cell exhaustion within a relatively uninflamed TME (Related to Figure 5). (**A**) The analysis of tumor-infiltrating immune cells in DLBCLs with *MAVS* high (50%) and low (50%). (**B**) The analysis of tumor-infiltrating immune cells in DLBCLs with *ADAR1* high (50%) and low (50%). Data was represented as mean +/- SD and p values were calculated using the Mann-Whitney U test.

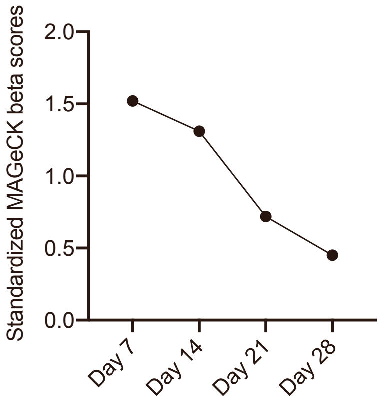

Figure S14. The dynamic effect of knocking out *MAVS* in RC-K8 cells (Related to Figure 4-5). Normalized MAGeCK score curves from a genome-wide screen in RC-K8 cells [3]. In a normalized, genome-wide screen such as this one, beta scores more than 3 or less than -3 are considered strongly significant. Based on these data, MAVS ablation has a “mild anti-proliferation effect,” which is most prominent on day 7 of the time course.

Table S2: Clinical characterization for DLBCL patients with high vs. low levels of ADAR1 isoforms ratio (p110/p150) (Related to Figure 1G)

	High	Low	*p* value*
**No. of patients**	27	27	
Age (years)			
**> 60**	11 (41%)	8 (30%)	0,393
**≤ 60**	16 (59%)	19 (70%)	
Gender			
**Female**	12 (44%)	7 (26%)	0,154
**Male**	15 (56%)	20 (74%)	
Performance status			
**0-1**	15 (56%)	19 (70%)	0,26
**2-4**	12 (44%)	8 (30%)	
Elevated LDH			
**Yes**	18 (67%)	11 (41%)	0,056
**No**	9 (33%)	16 (59%)	
Subtype			
**GCB**	7 (26%)	13 (48%)	0,091
**Non-GCB**	20 (74%)	14 (52%)	
Stage			
**I-II**	6 (22%)	13 (48%)	0,046
**III-IV**	21 (78%)	14 (52%)	
IPI			
**0-2**	14 (52%)	18 (67%)	0,169
**3-5**	13 (48%)	9 (33%)	

p value calculated based on chi-square probability test.

Table S4: Statistical analysis of editing/mutation exclusivity for the indicated pathways (Related to Figure 2)

Gene	Pathway	No. of samples with DNA mutations	No. of samples with RNA editing	No. of samples with both DNA mutations and RNA editing	No. of samples with either DNA mutations or RNA editing	Expected of samples with both DNA mutations and RNA editing by permutation	Expected of samples with either DNA mutations or RNA editing by permutation	P value of mutual exclusion*
**PRKCB**	BCR/NF-KB	7	35	2	38	6.154	29.691	2.60E-05
**BTK**	BCR/NF-KB	4	70	1	72	3.838	66.324	5.80E-05
**BCL2**	BCR/NF-KB	5	81	2	82	4.825	76.35	8.40E-05
**DDX3X**	RIG-I like receptor	7	8	1	13	4.056	6.889	0.001906
**TLR2**	BCR/NF-KB	2	25	0	27	1.852	23.296	0.002821
**PTEN**	TP53	3	35	1	36	2.841	32.317	0.004327
**MAPK8**	RIG-I like receptor	1	84	0	85	0.988	83.023	0.011644
**IGFBP3**	TP53	1	77	0	78	0.988	76.025	0.012403
**NLRX1**	RIG-I like receptor	1	44	0	45	0.978	43.044	0.022043
**REL**	BCR/NF-KB	1	44	0	45	0.978	43.044	0.022165
**SESN1**	TP53	1	31	0	32	0.969	30.062	0.031124

* p value was calculated based on Fisher's exact test.

Table S5: Clinical characterization for DLBCL patients with high vs. low level of MAVS expression (Related to Figure 5)

	**High**	**Low**	***p* value***
**No. of patients**	53	53	
Age (years)			
**> 60**	25 (47%)	31 (58%)	0,253
**≤ 60**	28 (55%)	22 (42%)	
Gender			
**Female**	22 (42%)	22 (42%)	1
**Male**	31 (58%)	31 (58%)	
Performance status			
**0-1**	38 (72%)	34 (64%)	0,405
**2-4**	15 (28%)	19 (36%)	
Elevated LDH			
**Yes**	24 (45%)	30 (57%)	0,244
**No**	29 (55%)	23 (43%)	
Subtype			
**GCB**	16 (31%)	18 (33%)	0,678
**Non-GCB**	20 (74%)	14 (52%)	
Stage			
**I-II**	23 (43%)	17 (32%)	0,229
**III-IV**	30 (57%)	36 (68%)	
IPI			
**0-2**	36 (68%)	25 (47%)	0,031
**3-5**	17 (32%)	28 (53%)	

* p value was calculated based on chi-square probability test.
The MAVS-high (top 50%) and -low (bottom 50%) expression groups are compared.

Table S6. Primers and oligos used in this study (Related to Figure 4)

#	Name	Sequence
**#1**	sgRNA1_F_exon3	caccGCTAGAGGAAGCCAAAGCCA
**#2**	sgRNA1_R_exon3	aaacTGGCTTTGGCTTCCTCTAGC
**#3**	sgRNA2_F_exon4	caccGGACAGGAGACGGAATTCGC
**#4**	sgRNA 2_R_exon4	aaacGCGAATTCCGTCTCCTGTCC
**#5**	non-targeting sgRNA_F	caccGTATTACTGATATTGGT
**#6**	non-targeting sgRNA_R	aaacACCAATATCAGTAATAC
**#7**	ADAR1ko_exon3	tatatatcttgtggaaaggacgaaaCACCGCTAGAGGAAGCCAAAGCCAgttttagagctagaaatagcaagttaaaa
**#8**	ADAR1ko_exon4	tatatatcttgtggaaaggacgaaaCACCGGACAGGAGACGGAATTCGCgttttagagctagaaatagcaagttaaaa
**#9**	non-targeting sgRNA (PX458)	tatatatcttgtggaaaggacgaaacaccGTATTACTGATATTGGTgttttagagctagaaatagcaagttaaaa
**#10**	ADAR1_gDNA_F	AAAGGACAGGCTGAGCCTTAAGGA
**#11**	ADAR1_gDNA_R	GCCATCTGCTTTGCCACTT
**#12**	MAVS_F	TACCCTGCCTGGCCTCAAACTATTA
**#13**	MAVS_R	ACTTCATGCTGTCTGGGAGCAA
**#14**	RIG-I_qPCR_F	GGTATAGAGTTACAGGCATTTC
**#15**	RIG-I_qPCR_R	TTGTTTACTAGTGTTGTGGC
**#16**	MAVS_qPCR_F	GAAATGAGGAGACCCCAG
**#17**	MAVS_qPCR_R	CAAGGCCCCTATTCTCAG
**#18**	PGK1_qPCR_F	CTAAGCAGATTGTGTGGAATG
**#19**	PGK1_qPCR_R	CTCACATGGCTGACTTTATC
**#20**	ISG15_qPCR_F	AGATCACCCAGAAGATCG
**#21**	ISG15_qPCR_R	TGTTATTCCTCACCAGGATG
**#22**	IFIH1_qPCR_F	GATTAAGTGGTGATACCCAAC
**#23**	IFIH1_qPCR_R	GTCTGACAATTGAACACCAG
**#24**	IRF9_qPCR_F	CCTGAGCCACAGGAAGTTACA
**#25**	IRF9_qPCR_R	CGCCCGTTGTAGATGAAGGT
**#26**	ISG20_qPCR_F	CAAGAGCATCCAGAACAG
**#27**	ISG20_qPCR_R	TAGTTGCTGTCCCAAAAAG
**#28**	IFNB1_qPCR_F	CGCCGCATTGACCATCTA
**#29**	IFNB1_qPCR_R	GACATTAGCCAGGAGGTTCTC
**#30**	B2M_qPCR_F	AAGGACTGGTCTTTCTATCTC
**#31**	B2M_qPCR_R	GATCCCACTTAACTATCTTGG
**#32**	BCL2L1_qPCR_F	ATCTCTTTCTCTCCCTTCAG

**#33**	BCL2L1_qPCR_R	CTTTCTGGGAAAGCTTGTAG
**#34**	CCL5_qPCR_F	AAGTCTCTAGGTTCTGAGC
**#35**	CCL5_qPCR_R	TTTTATGGTTGCATTGAGAAC
**#36**	SLC3A2_qPCR_F	TGTCATTCTGGACCTTACTC
**#37**	SLC3A2_qPCR_R	GATGCATCCTTCAGATTCTC
**#38**	SPP1_qPCR_F	GACCAAGGAAAACTCACTAC
**#39**	SPP1_qPCR_R	CTGTTTAACTGGTATGGCAC
**#40**	gRNA MAVS BB-455	5' Ts*G*sTs*AsAUCCCAG*C**C**A*CUUGA*G*A*sGsCsGsA – 3' -BG moiety
**#41**	gRNA MAVS BB-456	5' Cs*U*sCs*UsAACAAAA*A**C**A*CGAAA*AUsTsUsCsG – 3' -BG moiety
**#42**	gRNA MAVS NH-455	5' Ts*G*sTs*AsAUCCCAG*C**C**A*CUUGA*G*A*sGsCsGsA –Amino C6-3'
**#43**	gRNA MAVS NH-456	5' Cs*U*sCs*UsAACAAAA*A**C**A*CGAAA*AUsTsUsCsG – Amino C6-3'
**#44**	ADAR1_RT-PCR_F	CAGTTCGCTAGTCAAACCTGTGAGT
**#45**	ADAR1_RT-PCR_R	GTTACCTCTGTGAAACCCATGC

Oligos from #1-9 were used for cloning the gRNAs in the CRISPR/Cas9 plasmids. Lowercase letters represent the homology part needed for the cloning. Uppercase the sequence of the gRNA inserted either in LentiCRISPRv2 or in PX458. Oligos #10-39 were used for PCR amplification, sequencing and qRT-PCR analysis. Finally, #40-43 are the gRNAs used in the site directed RNA editing experiment. The C which creates the A:C mismatches is in bold. BG-conjugated gRNAs (#40 and #41) were synthesized and PAGE-purified from commercially acquired oligo nucleotides containing a 3'-amino-C6 linker (#42 and #43) (BioSpring, Germany) as described previously[4]. Nucleotides highlighted in red are unmodified and are placed opposite the triplet with the target A in the middle. Nucleotides highlighted in italic are modified with 2'-O-methylation. Nucleotides highlighted in blue are LNAs. The backbone contains terminal phosphorothioate linkages as indicated by "s". The last three nucleotides at the 3'-end are not complementary to the mRNA substrate but serve as linker sequence between gRNA and SNAP-tag. A Schematic of the binding of these gRNAs within MAVS 3'UTR is provided in Figure S9.

References

1. Kluesner, M., Tasakis, R.N., Lerner, T., Arnold, A., Wüst, S., Binder, M., Webber, B.R., Moriarity, B.S., and Pecori, R. (2021). MultiEditR: The first tool for detection and quantification of multiple RNA editing sites from Sanger sequencing demonstrates comparable fidelity to RNA-seq. Molecular Therapy - Nucleic Acids. 10.1016/j.omtn.2021.07.008.

2. Robinson, M.D., and Oshlack, A. (2010). A scaling normalization method for differential expression analysis of RNA-seq data. Genome Biology *11*, R25. 10.1186/gb-2010-11-3-r25.

3. Nie, M., Du, L., Ren, W., Joung, J., Ye, X., Shi, X., Ciftci, S., Liu, D., Wu, K., Zhang, F., et al. (2021). Genome-wide CRISPR screens reveal synthetic lethal interaction between CREBBP and EP300 in diffuse large B-cell lymphoma. Cell Death Dis *12*, 1–11. 10.1038/s41419-021-03695-8.

4. Hanswillemenke, A., Kuzdere, T., Vogel, P., Jékely, G., and Stafforst, T. (2015). Site-Directed RNA Editing in Vivo Can Be Triggered by the Light-Driven Assembly of an Artificial Riboprotein. Journal of the American Chemical Society. 10.1021/jacs.5b10216.
